# Reduced 3,4′-bi­pyrazoles carrying thio­phene and thia­zole substituents: structures of two intermediates and two products

**DOI:** 10.1107/S2056989021002310

**Published:** 2021-03-05

**Authors:** Chayanna Harish Chinthal, Hemmige S. Yathirajan, Nagaraja Manju, Balakrishna Kalluraya, Sabine Foro, Christopher Glidewell

**Affiliations:** a Department of Studies in Chemistry, University of Mysore, Manasagangotri, Mysuru-570 006, India; b Department of Studies in Chemistry, Mangalore University, Mangalagangotri, Mangalore-574 199, India; c Institute of Materials Science, Darmstadt University of Technology, Alarich-Weiss-Strasse 2, D-64287 Darmstadt, Germany; d School of Chemistry, University of St Andrews, St Andrews, Fife KY16 9ST, UK

**Keywords:** heterocyclic compounds, reduced bi­pyrazoles, synthesis, crystal structure, regiochemistry, hydrogen bonding, supra­molecular assembly

## Abstract

Cyclo­addition reactions between 3-(5-ar­yloxy-3-methyl-1-phenyl-1*H*-pyrazol-4-yl)-1-(thio­phen-2-yl)prop-2-en-1-ones and thio­semicarbazide leads to the formation of reduced 3,4′-bi­pyrazole-2-carbo­thio­amides. Further cyclo­addition of these inter­mediates with either diethyl acetyl­enedi­carboxyl­ate or 4-bromo­phenacyl bromide leads to reduced 3,4′-bi­pyrazoles carrying oxo­thia­zole or thia­zole substituents, respectively. The structures of two representative inter­mediates and two representative products established unambiguously the regiochemistry of the cyclo­addition reactions. The mol­ecules of 3′-methyl-5′-(2-methyl­phen­oxy)-1′-phenyl-5-(thio­phen-2-yl)-3,4-di­hydro-1′*H*,2*H*-3,4′-bi­pyra­zole-2-carbo­thio­amide, C_25_H_23_N_5_OS_2_ (Ia), are linked by N—H⋯N hydrogen bonds to form simple *C*(8) chains. The analogous compound 5′-(2,4-di­chloro­phen­oxy)-3′-methyl-1′-phenyl-5-(thio­phen-2-yl)-3,4-di­hydro-1′*H*,2*H*-3,4′-bi­pyra­zole-2-carbo­thio­amide hemihydrate crystallizes as a hemihydrate, C_24_H_19_Cl_2_N_5_OS_2_·0.5H_2_O (Ib), and the independent components are linked into a chain of spiro-fused *R* ^4^_4_(20) rings by a combination of O—H⋯N and N—H⋯O hydrogen bonds. In the structure of ethyl (*Z*)-2-{2-[3′-methyl-1′-phenyl-5-(thio­phen-2-yl)-5′-(2-methyl­phen­oxy)-3,4-di­hydro-1′*H*,2*H*-3,4′-bi­pyrazole-2-yl]-4-oxo-4,5-di­hydro­thia­zol-5-yl­idene}acetate, C_31_H_27_N_5_O_4_S_2_ (II), inversion-related pairs of mol­ecules are linked by paired C—H⋯π(arene) hydrogen bonds to form cyclic centrosymmetric dimers, but there are no direction-specific inter­molecular inter­actions in 4-(4-bromo­phen­yl)-2-[5′-(2,4-di­chloro­phen­oxy)-3′-methyl-1′-phenyl-5-(thio­phen-2-yl)-3,4-di­hydro-1′*H*,2*H*-3,4′-bi­pyrazole-2-yl]-4-thia­zole, C_32_H_22_BrCl_2_N_5_OS_2_ (III). Comparisons are made with the structures of some related compounds.

## Abstract 2

Reduced 3,4′-bi­pyrazole-2-carbo­thio­amides are formed in cyclo­addition reactions between chalcones and thio­semicarbazide, and these can undergo further cyclo­addition reactions to form oxothaazole of thia­zole substituents. Structure analysis establishes the regiochemistry of the cyclo­addition reactions and shows the very simple patterns of supra­molecular assembly in these compounds.

## Chemical context   

**Figure 1:**
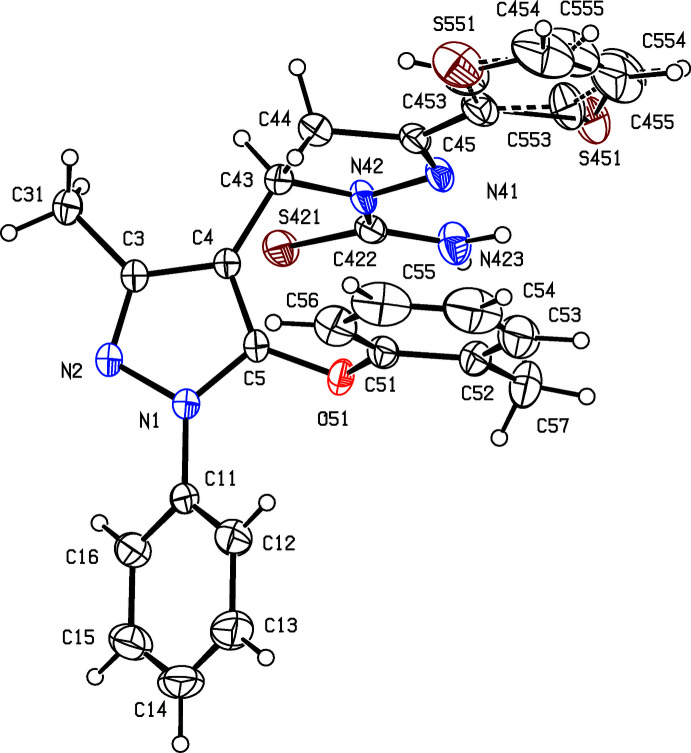
The mol­ecular structure of compound (Ia) showing the atom-labelling scheme and the disorder in the thio­phene unit, where the major disorder component is drawn using full lines and the minor disorder component is drawn using broken lines. Displacement ellipsoids are drawn at the 30% probability level.

**Figure 3:**
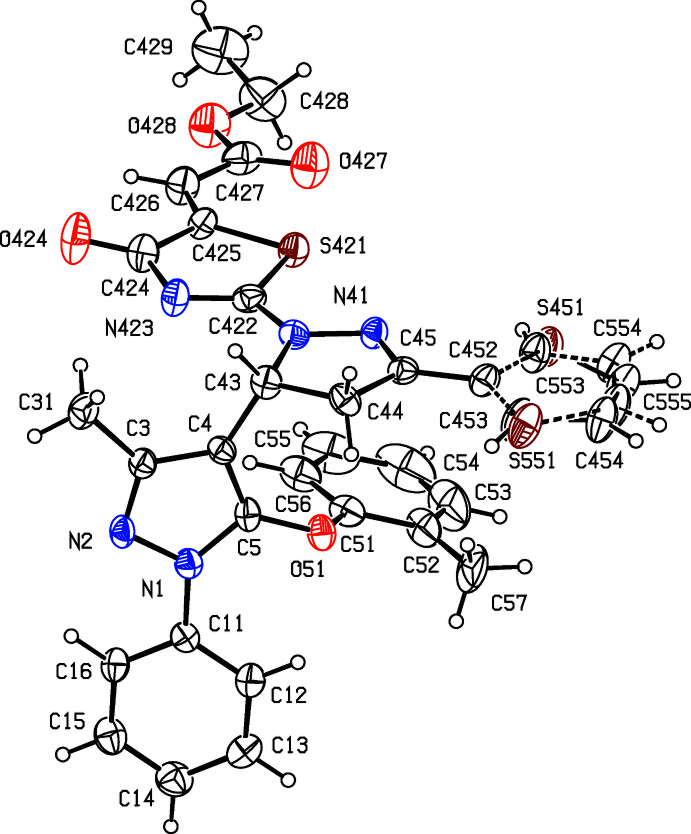
The mol­ecular structure of compound (II) showing the atom-labelling scheme and the disorder in the thio­phene unit, where the major disorder component is drawn using full lines and the minor disorder component is drawn using broken lines. Displacement ellipsoids are drawn at the 30% probability level.

**Figure 4:**
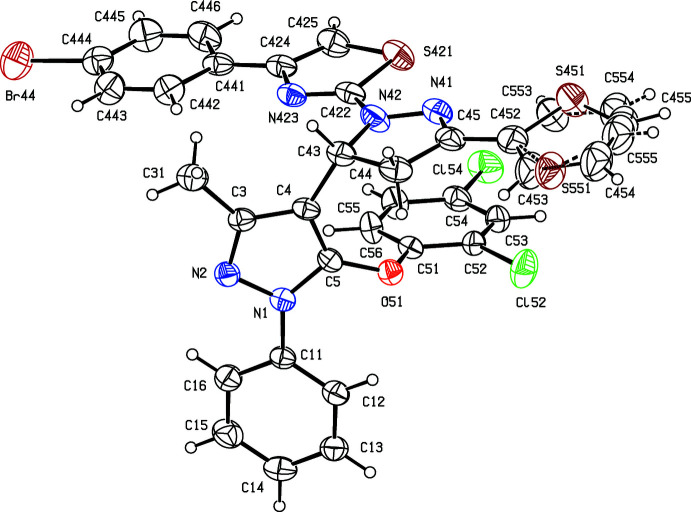
The mol­ecular structure of compound (III) showing the atom-labelling scheme and the disorder in the thio­phene unit, where the major disorder component is drawn using full lines and the minor disorder component is drawn using broken lines. Displacement ellipsoids are drawn at the 30% probability level.

Heterocyclic compounds containing the pyrazole unit have been found to exhibit a wide range of biological activities, including anti­bacterial and anti­fungal activity (Rai *et al.*, 2008 ▸; Isloor *et al.*, 2009 ▸; Vijesh *et al.*, 2013 ▸) and analgesic and anti-inflammatory activity (Girisha *et al.*, 2010 ▸; Isloor *et al.*, 2010 ▸; Vijesh *et al.*, 2013 ▸). It has also been found that the incorporation of a thia­zole or thia­zolone substituent often leads to enhanced activity (Sulthana *et al.*, 2015 ▸; Havrylyuk *et al.*, 2016 ▸), as does the incorporation of a thio­phene substituent (Rostom *et al.*, 2009 ▸; Bondock *et al.*, 2010 ▸). In this connection, a procedure has recently been developed (Manju *et al.*, 2019 ▸) for the synthesis of reduced 3,4′-bi­pyrazoles incorporating other heterocyclic units such as thia­zole, thia­zoline and thio­phene as integral components. In brief, condensation of a 5-ar­yloxy-3-methyl-1-phenyl-1*H*-pyrazole-4-carbaldehyde with 2-acetyl­thio­phene gives the corresponding chalcone (Shaibah *et al.*, 2020 ▸); chalcones of this type can undergo cyclo­condensation reactions with semicabazide to provide the inter­mediate carbo­thio­amides of type (I) (see Scheme). Further condensation of type (I) inter­mediates with diethyl acetyl­enedi­carboxyl­ate or with 4-bromo­phenacyl bromide gave the oxo­thia­zolyl­idene ester (II) or the thia­zole (III), respectively (see Scheme). Although the NMR spectra of the inter­mediates (I) and the products (II) and (III) contained all of the expected signals, it was not possible to establish uniquely from these data the regiochemistry of the cyclo­addition reactions leading to their formation, and accordingly we have determined the structures of two representative inter­mediates (Ia) and (Ib) (Figs. 1 ▸ and 2 ▸) and of two representative products (II) (Fig. 3 ▸) and (III) (Fig. 4 ▸).
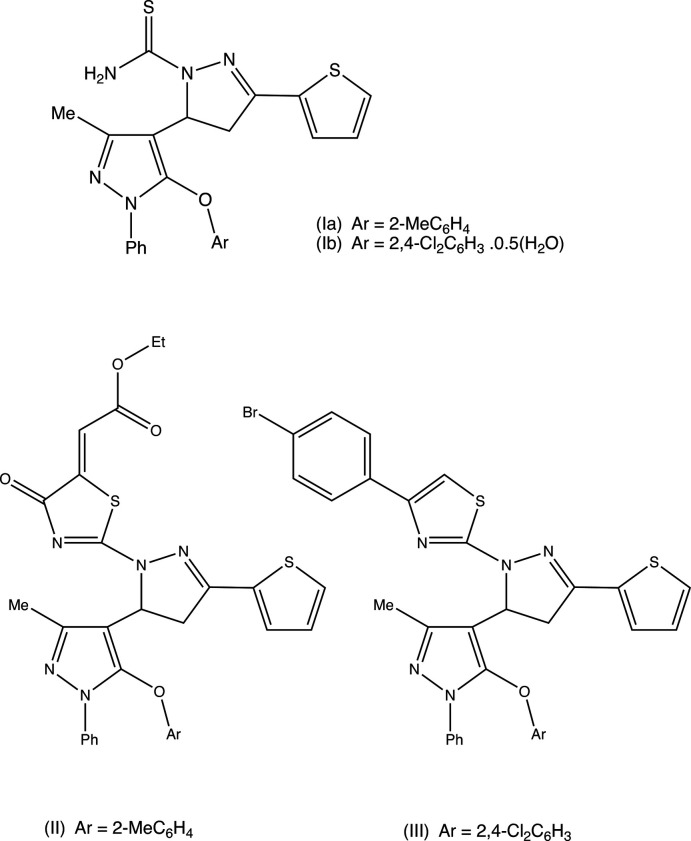


## Structural commentary   

**Figure 5:**
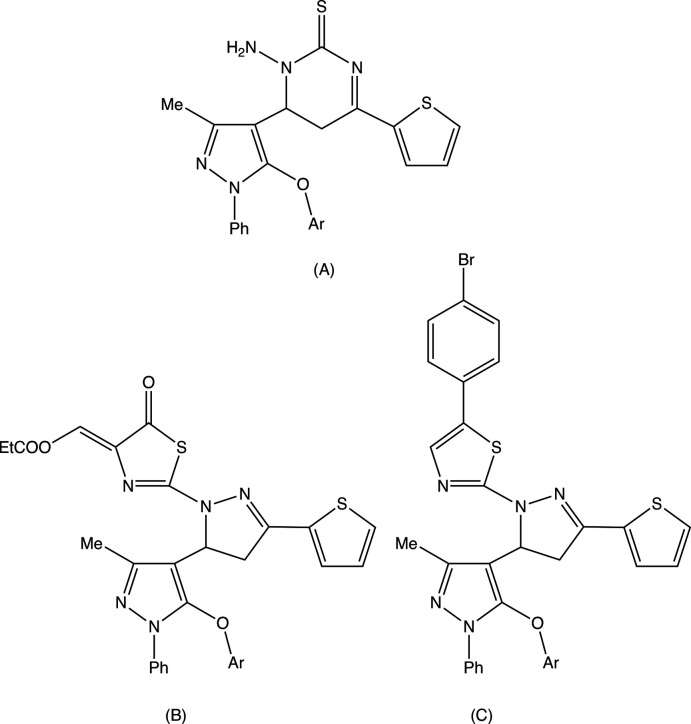
Possible regioisomers (A)–(C) of compounds (I)–(III), respectively.

Although compounds (Ia), (Ib), (II) and (III) were all crystallized under identical conditions, compound (Ib) crystallized as a hemihydrate, in which the water mol­ecules lies across a twofold rotation axis, while the other three compounds all crystallized in solvent-free form. In each compound, the thio­phene substituent is disordered over two sets of atomic sites (Section 6), whose relationship approximately corres­ponds to a rotation of 180° about the bond C45—C452 (Figs. 1 ▸–4 ▸ ▸ ▸). That the cyclo­condensation reactions between the chalcone precursors and thio­semicarbazide lead to the formation of new pyrazole rings indicates that it is the two N atoms of the hydrazine unit in thio­semicarbazide that participate in this reaction step. If the participants had been the two N atoms either side of the thio­carbonyl unit, then the products would have been the regioisomers of type (A), containing a newly formed reduced pyrimidine ring in place of the pyrazole ring actually observed (Fig. 5 ▸). Similarly, in the cyclo­condensation reactions between the carbo­thio­amides (I) and either diethyl acetyl­enedi­carboxyl­ate or 4-bromo­phenacyl bromide to form (II) and (III), respectively, alternative regiochemistry is possible in each case, to yield products of types (B) and (C), respectively (Fig. 5 ▸). The X-ray analyses reported here have confirmed that the single products formed in each of these cyclo­condensation reactions (Manju *et al.*, 2019 ▸) have structures of types (I)–(III), as opposed to the possible alternative isomers (A)–(C).

## Supra­molecular features   

**Figure 6:**
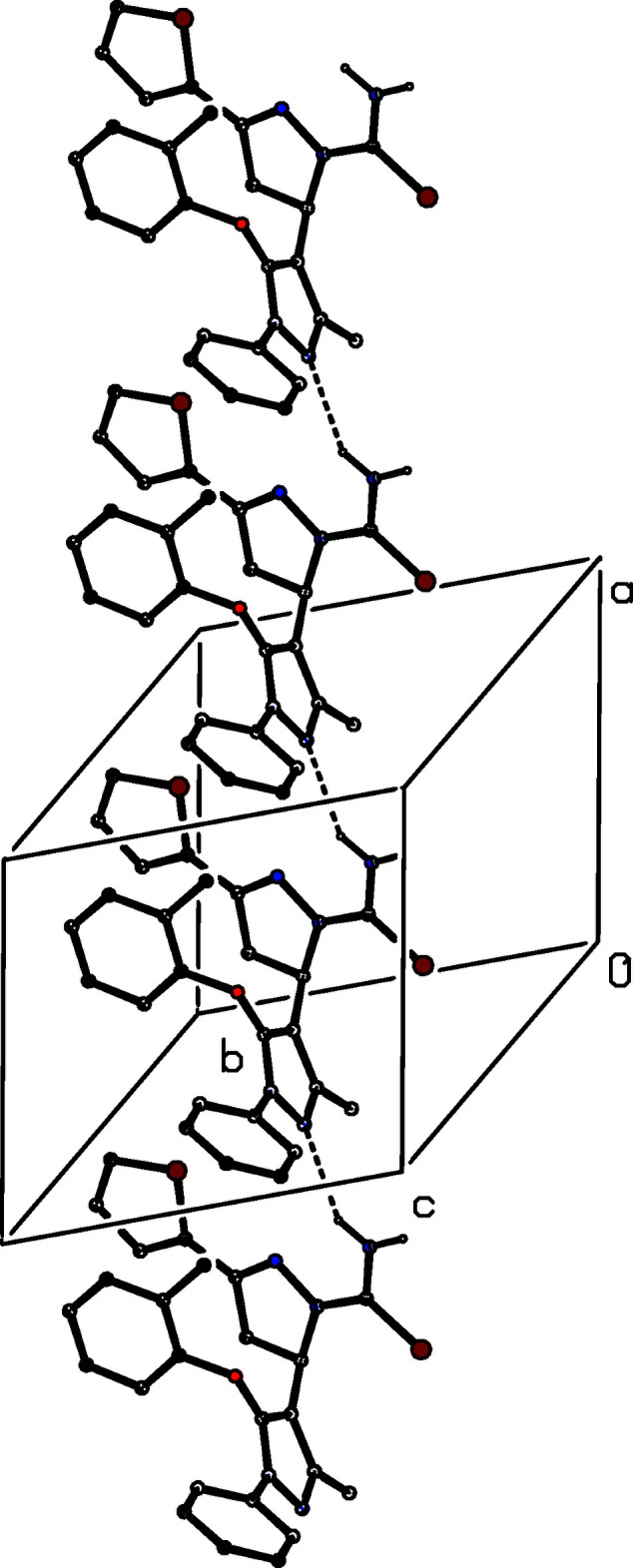
Part of the crystal structure of compound (Ia) showing the formation of a hydrogen-bonded chain running parallel to the [100] direction. Hydrogen bonds are drawn as dashed lines and, for the sake of clarity, the H atoms which are bonded to C atoms have been omitted.

The supra­molecular assembly of compound (Ia) is extremely simple: a single N—H⋯N hydrogen bond (Table 1 ▸) links mol­ecules that are related by translation into a *C*(8) (Etter, 1990 ▸; Etter *et al.*, 1990 ▸; Bernstein *et al.*, 1995 ▸) chain running parallel to the [100] direction (Fig. 6 ▸), but there are no direction-specific inter­actions between adjacent chains.

**Figure 7:**
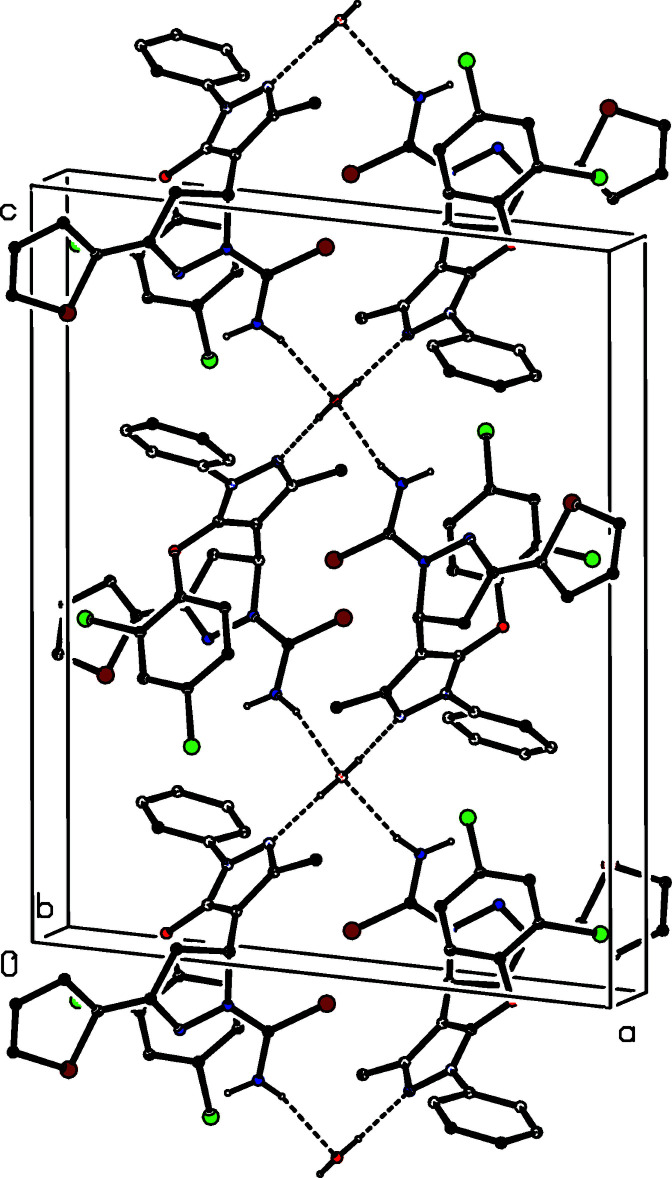
Part of the crystal structure of compound (Ib) showing the formation of a hydrogen-bonded chain of spiro-fused rings running parallel to the [001] direction. Hydrogen bonds are drawn as dashed lines and, for the sake of clarity, the H atoms which are bonded to C atoms have been omitted.

Compound (Ib) is a hemihydrate in which the water component lies across a twofold rotation axis, and the supra­molecular aggregation is more complex than that in (Ia). There is an O—H⋯N hydrogen bond within the selected asymmetric unit (Table 1 ▸), and pairs of inversion-related bimolecular units of this type are linked by paired N—H⋯O hydrogen bonds to form an 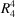
(20) ring. Propagation of this motif by the action of the twofold rotation axes generates a chain of spiro-fused 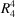
(20) rings running parallel to the [001] direction, in which the centrosymmetric rings are centred at (0.5, 0.5, 0.5*n*) where *n* represents an integer (Fig. 7 ▸). Within this chain the water mol­ecules, which act as double donors in O—H⋯N hydrogen bonds and double acceptors in N—H⋯O hydrogen bonds, are the points of fusion between adjacent rings (Fig. 7 ▸).

**Figure 8:**
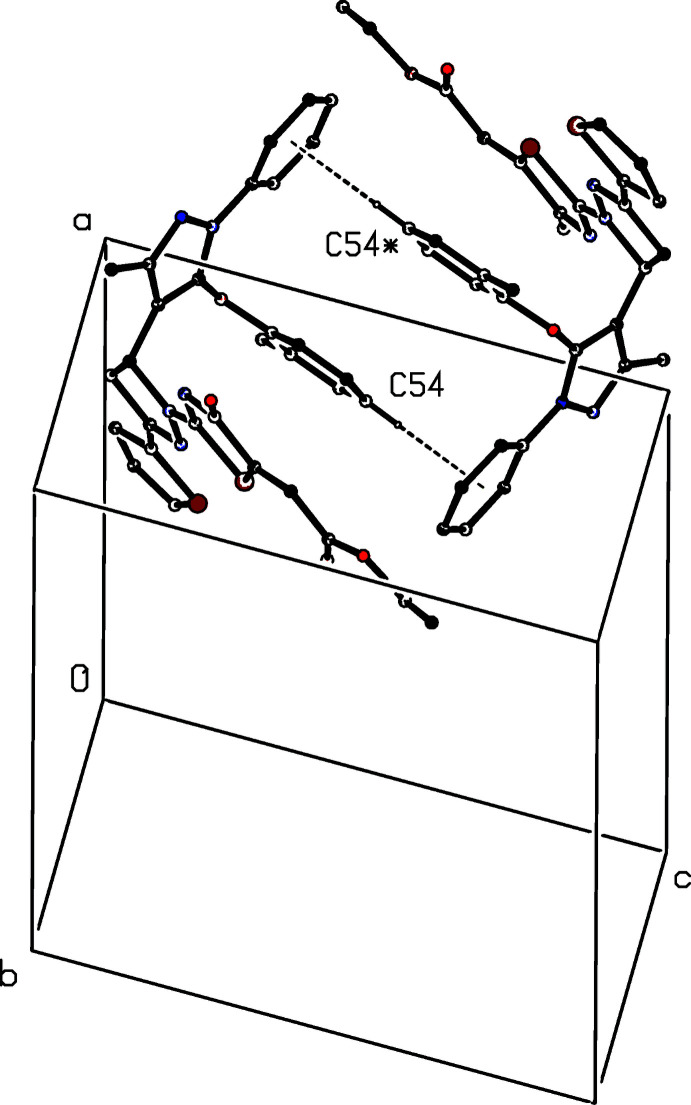
Part of the crystal structure of compound (II) showing the formation of a cyclic centrosymmetric dimer containing C—H⋯π(arene) hydrogen bonds. For the sake of clarity, the minor disorder components, and the H atoms not involved in the motif shown have been omitted. The atom marked with an asterisk (*) is at the symmetry position (2 − *x*, −*y*, 1 − *z*).

There are three short inter­molecular contacts in the structure of compound (II). That involving atom C13 (Table 1 ▸) has a very small *D*—H⋯*A* angle, and so is unlikely to be structurally significant (Wood *et al.*, 2009 ▸), while that involving atom C553 applies only to the minor disorder component, and is absent for the majority of the mol­ecules. The only possible significant inter­action is thus that involving atom C54, which links inversion-related pairs of mol­ecules to form a cyclic centrosymmetric motif (Fig. 8 ▸). There are no significant hydrogen bonds of any type in the structure of compound (III).

## Database survey   

Structures have been reported recently for a number of compounds related to those reported here, including precursors and inter­mediates in the synthetic pathways to compounds (I)–(III). The structures of five examples of 5-ar­yloxy-3-methyl-1-phenyl-1*H*-pyrazole-4-carbaldehydes have been reported (Shahani *et al.*, 2011 ▸; Vinutha *et al.*, 2014 ▸; Glidewell *et al.*, 2019 ▸; Kiran Kumar *et al.*, 2019 ▸), as have those (Shaibah *et al.*, 2020 ▸) of two isostructural chalcones derived from two such carbaldehydes by condensation reactions with 2-acetyl­thio­phene, in each of which the thio­phene unit shows the same type of disorder as observed here in compounds (Ia), (Ib), (II) and (III). Structures have also been reported (Cuartas *et al.*, 2017 ▸; Kiran Kumar *et al.*, 2019 ▸) for several reduced 3,4′-bi­pyrazoles formed by cyclo­condensation reactions between chalcones and hydrazine followed by N-acetyl­ation. However, the only structure reported to date of a product in which the 3,4′-bi­pyrazole unit is embedded within a group of other cyclic substituents, as in (I)–(III) is that for the methyl ester analogue of (II) (Manju *et al.*, 2019 ▸). The original report on this compound provided no crystallographic information other than a mol­ecular structure plot. However, the deposited CIF (CCDC deposition No. 1588961) shows that the reflection data have been subjected to the SQUEEZE procedure (Spek, 2015 ▸), although this is not mentioned in the original report. The CIF also shows two sites for the O atom of the ar­yloxy unit, *ca* 1.28 Å apart with occupancies of 0.843 (6) and 0.157 (6) and involving some unexpected geometrical features, although all other atoms are reported as being fully ordered. Hence this structure is unlikely to be entirely correct.

## Synthesis and crystallization   

Samples of compounds (Ia), (Ib), (II) and (III) were prepared using the methods previously reported (Manju *et al.*, 2019 ▸). Crystals suitable for single-crystal X-ray diffraction were grown by slow evaporation, at ambient temperature and in the presence of air, of solutions in a mixture of ethanol and *N*,*N*-di­methyl­formamide (initial composition 3:1, *v*/*v*).

## Refinement   

Crystal data, data collection and structure refinement details are summarized in Table 2 ▸. Several bad outlier reflections were omitted from the refinements. *i.e*. for (Ia) (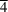,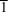,18); for (Ic) (1,1,1), (14,0,0), (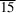,0,6), (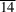,1,19), (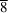,9,7) and (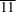,3,2); and for (II) (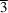,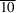,2) and (0,5,13). All H atoms, apart from those in the minor disorder components, were located in difference maps. The H atoms bonded to C atoms were then treated as riding atoms in geometrically idealized positions with C—H distances of 0.93 Å (alkenyl, aromatic and thien­yl), 0.96 Å (CH_3_), 0.97 Å (CH_2_) or 0.98 Å (aliphatic C—H), and with *U*_iso_(H) = *kU*_eq_(C), where *k* = 1.5 for the methyl groups, which were permitted to rotate but not to tilt, and 1.2 for all other H atoms bonded to C atoms. For the H atoms bonded to N or O atoms, the atomic coordinates were refined with *U*_iso_(H) = 1.2U_eq_(N) or 1.5*U*_eq_(O), giving the N—H and O—H distances shown in Table 1 ▸. For the minor disorder components, the bonded distances and the 1,3 non-bonded distances were restrained to be the same as the corresponding distances in the major disorder components, subject to s.u. values of 0.01 and 0.02 Å, respectively. In addition, the anisotropic displace­ment parameters associated with pairs of atomic sites occupying essentially the same regions of physical space were constrained to be equal. Subject to these conditions, the occupancies, in the crystals selected for data collection, of the disordered thienyl units refined to 0.866 (3) and 0.134 (3) in (Ia), 0.951 (3) and 0.049 (3) in (Ib), 0.768 (6) and 0.232 (6) in (II), and 0.947 (4) and 0.053 (4) in (III).

## Supplementary Material

Crystal structure: contains datablock(s) global, Ia, Ib, II, III. DOI: 10.1107/S2056989021002310/dx2035sup1.cif

Structure factors: contains datablock(s) Ia. DOI: 10.1107/S2056989021002310/dx2035Iasup2.hkl

Supporting information file. DOI: 10.1107/S2056989021002310/dx2035Iasup6.cml

Structure factors: contains datablock(s) Ib. DOI: 10.1107/S2056989021002310/dx2035Ibsup3.hkl

Supporting information file. DOI: 10.1107/S2056989021002310/dx2035Ibsup7.cml

Structure factors: contains datablock(s) II. DOI: 10.1107/S2056989021002310/dx2035IIsup4.hkl

Supporting information file. DOI: 10.1107/S2056989021002310/dx2035IIsup8.cml

Structure factors: contains datablock(s) III. DOI: 10.1107/S2056989021002310/dx2035IIIsup5.hkl

CCDC references: 2065478, 2065477, 2065476, 2065475

Additional supporting information:  crystallographic information; 3D view; checkCIF report

## Figures and Tables

**Figure 2:**
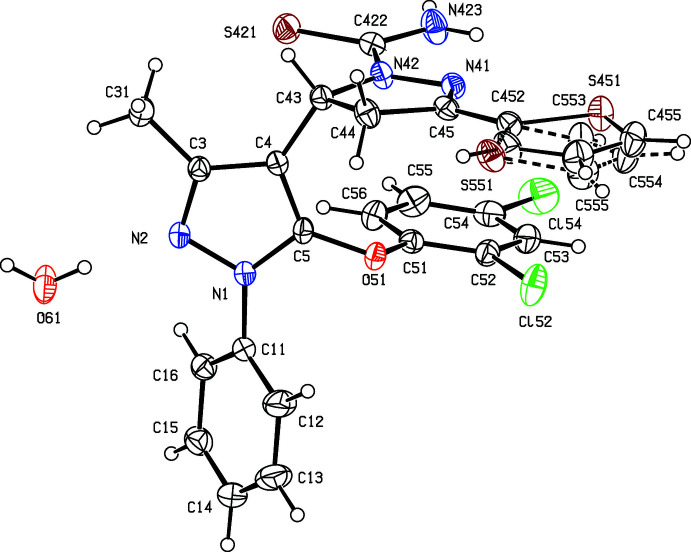
The structure of the independent components in compound (Ib) showing the atom-labelling scheme and the disorder in the thio­phene unit, where the major disorder component is drawn using full lines and the minor disorder component is drawn using broken lines. The water mol­ecule lies across a twofold rotation axis and the displacement ellipsoids are drawn at the 30% probability level.

**Table 1:** Hydrogen bonds and short inter- and intra­molecular contacts (Å, °) *Cg*1 represents the centroid of the C11–C16 ring.

Compound	*D*—H⋯*A*	*D*—H	H⋯*A*	*D*⋯*A*	*D*—H⋯*A*
(Ia)	N423—H42*A*⋯N2^i^	0.92 (3)	2.28 (3)	3.111 (3)	150 (2)
	N423—H42*A*⋯N41	0.92 (3)	2.27 (3)	2.628 (4)	103 (2)
(Ib)	O61—H61⋯N2	0.88 (3)	2.03 (3)	2.900 (2)	176 (2)
	N423—H42*A*⋯O61^ii^	0.84 (3)	2.33 (3)	3.154 (3)	167 (3)
	N423—H42*B*⋯N41	0.85 (3)	2.27 (3)	2.648 (3)	107 (2)
(II)	C13—H13⋯O424^iii^	0.93	2.49	3.200 (7)	133
	C54—H54⋯*Cg*1^iv^	0.93	2.91	3.714 (12)	146
	C553—H553⋯*Cg*1^v^	0.93	2.92	3.76 (5)	151

Symmetry codes: (i) 1 + *x*, *y*, *z*; (ii) 1 − *x*, 1 − *y*, 1 − *z*; (iii) *x*, −1 + *y*, *z*; (iv) 2 − *x*, −*y*, 1 − *z*; (v) −1 + *x*, *y*, *z*.

**Table 2:** Experimental details

	(Ia)	(Ib)	(II)	(III)
Crystal data				
Chemical formula	C_25_H_23_N_5_OS_2_	2C_24_H_19_Cl_2_N_5_OS_2_·H_2_O	C_31_H_27_N_5_O_4_S_2_	C_32_H_22_BrCl_2_N_5_OS_2_
*M*_r_	473.60	1074.94	597.69	707.47
Crystal system, space group	Triclinic, *P*\overline{1}	Monoclinic, *P*2/*c*	Triclinic, *P*\overline{1}	Triclinic, *P*\overline{1}
Temperature (K)	296	296	296	296
*a*, *b*, *c* (Å)	8.6269 (7), 9.8418 (9), 14.900 (1)	15.037 (1), 8.4266 (6), 19.471 (1)	10.783 (2), 11.683 (3), 13.577 (3)	12.3200 (9), 12.5700 (9), 12.7742 (9)
α, β, γ (°)	90.588 (7), 106.162 (8), 101.441 (7)	90, 96.246 (6), 90	93.54 (2), 105.17 (2), 113.20 (2)	117.202 (8), 102.879 (7), 105.727 (7)
*V* (Å^3^)	1188.05 (17)	2452.5 (3)	1490.9 (6)	1548.4 (2)
*Z*	2	2	2	2
Radiation type	Mo *K*α	Mo *K*α	Mo *K*α	Mo *K*α
μ (mm^−1^)	0.25	0.47	0.22	1.67
Crystal size (mm)	0.36 × 0.12 × 0.04	0.36 × 0.12 × 0.12	0.48 × 0.12 × 0.06	0.40 × 0.40 × 0.08

Data collection				
Diffractometer	Oxford Diffraction Xcalibur with Sapphire CCD detector	Oxford Diffraction Xcalibur with Sapphire CCD detector	Oxford Diffraction Xcalibur with Sapphire CCD detector	Oxford Diffraction Xcalibur with Sapphire CCD detector
Absorption correction	Multi-scan (*CrysAlis RED*; Oxford Diffraction, 2009 ▸)	Multi-scan (*CrysAlis RED*; Oxford Diffraction, 2009 ▸)	Multi-scan (*CrysAlis RED*; Oxford Diffraction, 2009 ▸)	Multi-scan (*CrysAlis RED*; Oxford Diffraction, 2009 ▸)
*T*_min_, *T*_max_	0.949, 0.990	0.922, 0.946	0.849, 0.987	0.779, 0.875
No. of measured, independent and observed [*I* > 2σ(*I*)] reflections	8241, 4898, 2524	10494, 5290, 3075	10433, 5561, 1730	10549, 5773, 3158
*R*_int_	0.030	0.033	0.138	0.022
(sin θ/λ)_max_ (Å^−1^)	0.629	0.651	0.607	0.607

Refinement				
*R*[*F* ^2^ > 2σ(*F* ^2^)], *wR*(*F* ^2^), *S*	0.056, 0.112, 1.03	0.046, 0.097, 0.98	0.079, 0.143, 0.88	0.039, 0.095, 0.93
No. of reflections	4898	5290	5561	5773
No. of parameters	319	335	395	402
No. of restraints	10	10	10	10
H-atom treatment	H atoms treated by a mixture of independent and constrained refinement	H atoms treated by a mixture of independent and constrained refinement	H-atom parameters constrained	H-atom parameters constrained
Δρ_max_, Δρ_min_ (e Å^−3^)	0.18, −0.18	0.26, −0.30	0.26, −0.24	0.47, −0.34

Computer programs: *CrysAlis CCD* and *CrysAlis RED* (Oxford Diffraction, 2009 ▸), *SHELXT* (Sheldrick, 2015*a* ▸), *SHELXL2014* (Sheldrick, 2015*b* ▸) and *PLATON* (Spek, 2020 ▸).

## supplementary crystallographic information

### 3'-Methyl-5'-(2-methylphenoxy)-1'-phenyl-5-(thiophen-2-yl)-3,4-dihydro-1'H,2H-3,4'-bipyrazole-2-carbothioamide (Ia) Crystal data

**Table:**

C_25_H_23_N_5_OS_2_	*Z* = 2
*M**_r_* = 473.60	*F*(000) = 496
Triclinic, *P*1	*D*_x_ = 1.324 Mg m^−^^3^
*a* = 8.6269 (7) Å	Mo *K*α radiation, λ = 0.71073 Å
*b* = 9.8418 (9) Å	Cell parameters from 5098 reflections
*c* = 14.900 (1) Å	θ = 2.9–27.9°
α = 90.588 (7)°	µ = 0.25 mm^−^^1^
β = 106.162 (8)°	*T* = 296 K
γ = 101.441 (7)°	Needle, yellow
*V* = 1188.05 (17) Å^3^	0.36 × 0.12 × 0.04 mm

### 3'-Methyl-5'-(2-methylphenoxy)-1'-phenyl-5-(thiophen-2-yl)-3,4-dihydro-1'H,2H-3,4'-bipyrazole-2-carbothioamide (Ia) Data collection

**Table:**

Oxford Diffraction Xcalibur with Sapphire CCD detector diffractometer	4898 independent reflections
Radiation source: Enhance (Mo) X-ray Source	2524 reflections with *I* > 2σ(*I*)
Graphite monochromator	*R*_int_ = 0.030
ω scans	θ_max_ = 26.6°, θ_min_ = 2.9°
Absorption correction: multi-scan (CrysAlis RED; Oxford Diffraction, 2009)	*h* = −10→9
*T*_min_ = 0.949, *T*_max_ = 0.990	*k* = −10→12
8241 measured reflections	*l* = −16→18

### 3'-Methyl-5'-(2-methylphenoxy)-1'-phenyl-5-(thiophen-2-yl)-3,4-dihydro-1'H,2H-3,4'-bipyrazole-2-carbothioamide (Ia) Refinement

**Table:**

Refinement on *F*^2^	Primary atom site location: difference Fourier map
Least-squares matrix: full	Hydrogen site location: mixed
*R*[*F*^2^ > 2σ(*F*^2^)] = 0.056	H atoms treated by a mixture of independent and constrained refinement
*wR*(*F*^2^) = 0.112	*w* = 1/[σ^2^(*F*_o_^2^) + (0.038*P*)^2^ + 0.0763*P*] where *P* = (*F*_o_^2^ + 2*F*_c_^2^)/3
*S* = 1.03	(Δ/σ)_max_ < 0.001
4898 reflections	Δρ_max_ = 0.18 e Å^−^^3^
319 parameters	Δρ_min_ = −0.18 e Å^−^^3^
10 restraints	

### 3'-Methyl-5'-(2-methylphenoxy)-1'-phenyl-5-(thiophen-2-yl)-3,4-dihydro-1'H,2H-3,4'-bipyrazole-2-carbothioamide (Ia) Special details

**Table:**

Experimental. CrysAlis RED, Oxford Diffraction Ltd., 2009 Empirical absorption correction using spherical harmonics, implemented in SCALE3 ABSPACK scaling algorithm.
Geometry. All esds (except the esd in the dihedral angle between two l.s. planes) are estimated using the full covariance matrix. The cell esds are taken into account individually in the estimation of esds in distances, angles and torsion angles; correlations between esds in cell parameters are only used when they are defined by crystal symmetry. An approximate (isotropic) treatment of cell esds is used for estimating esds involving l.s. planes.

### 3'-Methyl-5'-(2-methylphenoxy)-1'-phenyl-5-(thiophen-2-yl)-3,4-dihydro-1'H,2H-3,4'-bipyrazole-2-carbothioamide (Ia) Fractional atomic coordinates and isotropic or equivalent isotropic displacement parameters (Å^2^)

**Table:**

	*x*	*y*	*z*	*U*_iso_*/*U*_eq_	Occ. (<1)
N1	−0.0795 (2)	0.6663 (2)	0.30851 (14)	0.0406 (6)	
N2	−0.2192 (2)	0.6179 (2)	0.23518 (15)	0.0451 (6)	
C3	−0.1670 (3)	0.6245 (3)	0.15926 (18)	0.0421 (7)	
C4	0.0047 (3)	0.6737 (3)	0.18050 (17)	0.0384 (7)	
C5	0.0541 (3)	0.6994 (3)	0.27513 (17)	0.0384 (7)	
C11	−0.0885 (3)	0.6605 (3)	0.40219 (18)	0.0445 (7)	
C12	0.0024 (3)	0.7654 (4)	0.4690 (2)	0.0625 (9)	
H12	0.0687	0.8426	0.4532	0.075*	
C13	−0.0067 (4)	0.7541 (5)	0.5603 (2)	0.0816 (12)	
H13	0.0563	0.8232	0.6063	0.098*	
C14	−0.1073 (5)	0.6421 (5)	0.5835 (2)	0.0838 (12)	
H14	−0.1123	0.6354	0.6448	0.101*	
C15	−0.2004 (4)	0.5399 (4)	0.5157 (3)	0.0757 (11)	
H15	−0.2704	0.4648	0.5310	0.091*	
C16	−0.1904 (3)	0.5484 (3)	0.4250 (2)	0.0565 (8)	
H16	−0.2524	0.4784	0.3793	0.068*	
C31	−0.2869 (3)	0.5831 (3)	0.06491 (18)	0.0615 (9)	
H31A	−0.2770	0.6581	0.0249	0.092*	
H31B	−0.3970	0.5616	0.0707	0.092*	
H31C	−0.2637	0.5027	0.0384	0.092*	
N41	0.4015 (2)	0.7247 (3)	0.16104 (14)	0.0415 (6)	
N42	0.2565 (2)	0.6253 (2)	0.14876 (14)	0.0393 (6)	
C43	0.1056 (3)	0.6826 (3)	0.11269 (17)	0.0412 (7)	
H43	0.0379	0.6306	0.0538	0.049*	
C44	0.1805 (3)	0.8295 (3)	0.09133 (18)	0.0496 (8)	
H44A	0.1515	0.8403	0.0244	0.060*	
H44B	0.1441	0.8997	0.1218	0.060*	
C45	0.3626 (3)	0.8383 (3)	0.13106 (18)	0.0418 (7)	
S421	0.09307 (9)	0.36681 (8)	0.14413 (5)	0.0559 (3)	
C422	0.2638 (3)	0.4938 (3)	0.16724 (17)	0.0408 (7)	
N423	0.4148 (3)	0.4679 (3)	0.20351 (18)	0.0538 (7)	
H42A	0.506 (3)	0.539 (3)	0.2180 (18)	0.065*	
H42B	0.426 (3)	0.380 (3)	0.2139 (19)	0.065*	
S451	0.69257 (13)	0.96287 (15)	0.18083 (12)	0.0769 (5)	0.866 (3)
C452	0.4879 (3)	0.9623 (3)	0.13580 (19)	0.0515 (8)	0.866 (3)
C453	0.4652 (18)	1.0876 (10)	0.1098 (14)	0.0769 (15)	0.866 (3)
H453	0.3615	1.1067	0.0830	0.092*	0.866 (3)
C454	0.6146 (9)	1.1897 (8)	0.1270 (14)	0.104 (2)	0.866 (3)
H454	0.6204	1.2821	0.1127	0.125*	0.866 (3)
C455	0.7456 (8)	1.1343 (6)	0.1666 (8)	0.099 (2)	0.866 (3)
H455	0.8539	1.1848	0.1844	0.119*	0.866 (3)
S551	0.456 (3)	1.1213 (19)	0.104 (3)	0.0769 (15)	0.134 (3)
C552	0.4879 (3)	0.9623 (3)	0.13580 (19)	0.0515 (8)	0.134 (3)
C553	0.6476 (19)	0.960 (4)	0.159 (4)	0.0769 (5)	0.134 (3)
H553	0.6877	0.8792	0.1740	0.092*	0.134 (3)
C554	0.751 (3)	1.092 (4)	0.158 (6)	0.099 (2)	0.134 (3)
H554	0.8660	1.1083	0.1735	0.119*	0.134 (3)
C555	0.661 (4)	1.189 (4)	0.133 (10)	0.104 (2)	0.134 (3)
H555	0.7062	1.2828	0.1317	0.125*	0.134 (3)
O51	0.20838 (18)	0.73742 (19)	0.33641 (11)	0.0434 (5)	
C51	0.2920 (3)	0.8764 (3)	0.34104 (17)	0.0432 (7)	
C52	0.4615 (3)	0.8993 (4)	0.37778 (18)	0.0535 (8)	
C53	0.5473 (5)	1.0365 (5)	0.3857 (2)	0.0833 (13)	
H53	0.6615	1.0564	0.4102	0.100*	
C54	0.4682 (7)	1.1423 (5)	0.3583 (3)	0.1011 (15)	
H54	0.5291	1.2330	0.3649	0.121*	
C55	0.3006 (6)	1.1174 (4)	0.3213 (3)	0.0882 (12)	
H55	0.2478	1.1901	0.3018	0.106*	
C56	0.2104 (4)	0.9822 (4)	0.3131 (2)	0.0626 (9)	
H56	0.0961	0.9634	0.2889	0.075*	
C57	0.5475 (4)	0.7841 (4)	0.4051 (2)	0.0804 (11)	
H57A	0.6602	0.8211	0.4398	0.121*	
H57B	0.5441	0.7310	0.3500	0.121*	
H57C	0.4940	0.7253	0.4435	0.121*	

### 3'-Methyl-5'-(2-methylphenoxy)-1'-phenyl-5-(thiophen-2-yl)-3,4-dihydro-1'H,2H-3,4'-bipyrazole-2-carbothioamide (Ia) Atomic displacement parameters (Å^2^)

**Table:**

	*U*^11^	*U*^22^	*U*^33^	*U*^12^	*U*^13^	*U*^23^
N1	0.0280 (12)	0.0587 (17)	0.0333 (13)	0.0070 (10)	0.0073 (10)	0.0036 (11)
N2	0.0269 (12)	0.0642 (18)	0.0403 (13)	0.0050 (11)	0.0063 (11)	0.0041 (11)
C3	0.0276 (14)	0.057 (2)	0.0382 (16)	0.0036 (12)	0.0073 (12)	0.0040 (13)
C4	0.0265 (14)	0.0520 (19)	0.0335 (16)	0.0026 (12)	0.0072 (12)	0.0031 (13)
C5	0.0237 (14)	0.0491 (19)	0.0400 (16)	0.0047 (12)	0.0073 (12)	0.0048 (13)
C11	0.0319 (15)	0.070 (2)	0.0363 (17)	0.0195 (14)	0.0112 (13)	0.0073 (15)
C12	0.0516 (19)	0.089 (3)	0.049 (2)	0.0129 (17)	0.0179 (16)	−0.0042 (18)
C13	0.071 (2)	0.129 (4)	0.049 (2)	0.029 (2)	0.0175 (19)	−0.011 (2)
C14	0.085 (3)	0.143 (4)	0.047 (2)	0.059 (3)	0.031 (2)	0.023 (2)
C15	0.073 (2)	0.105 (3)	0.069 (2)	0.037 (2)	0.038 (2)	0.039 (2)
C16	0.0508 (18)	0.072 (2)	0.053 (2)	0.0205 (16)	0.0208 (15)	0.0169 (16)
C31	0.0324 (16)	0.095 (3)	0.0440 (18)	−0.0081 (15)	0.0050 (14)	−0.0017 (17)
N41	0.0288 (12)	0.0448 (16)	0.0471 (14)	−0.0054 (11)	0.0141 (10)	−0.0002 (12)
N42	0.0259 (12)	0.0429 (16)	0.0468 (14)	−0.0018 (10)	0.0132 (10)	0.0027 (11)
C43	0.0313 (15)	0.054 (2)	0.0342 (15)	0.0018 (13)	0.0073 (12)	0.0049 (13)
C44	0.0437 (17)	0.060 (2)	0.0465 (17)	0.0070 (14)	0.0172 (14)	0.0109 (15)
C45	0.0369 (16)	0.049 (2)	0.0376 (16)	−0.0023 (14)	0.0154 (13)	0.0021 (14)
S421	0.0477 (5)	0.0563 (6)	0.0525 (5)	−0.0151 (4)	0.0143 (4)	0.0049 (4)
C422	0.0387 (17)	0.049 (2)	0.0340 (16)	−0.0004 (14)	0.0155 (13)	0.0007 (13)
N423	0.0410 (15)	0.0452 (18)	0.0733 (18)	0.0051 (13)	0.0161 (14)	0.0093 (15)
S451	0.0458 (7)	0.0803 (9)	0.0897 (12)	−0.0215 (6)	0.0194 (7)	0.0077 (7)
C452	0.0507 (18)	0.050 (2)	0.0530 (19)	−0.0039 (15)	0.0241 (15)	0.0042 (15)
C453	0.095 (3)	0.050 (5)	0.095 (3)	0.014 (4)	0.041 (2)	0.022 (5)
C454	0.144 (6)	0.050 (3)	0.120 (5)	−0.023 (4)	0.071 (7)	0.013 (3)
C455	0.097 (3)	0.076 (5)	0.106 (4)	−0.048 (3)	0.046 (3)	−0.013 (5)
S551	0.095 (3)	0.050 (5)	0.095 (3)	0.014 (4)	0.041 (2)	0.022 (5)
C552	0.0507 (18)	0.050 (2)	0.0530 (19)	−0.0039 (15)	0.0241 (15)	0.0042 (15)
C553	0.0458 (7)	0.0803 (9)	0.0897 (12)	−0.0215 (6)	0.0194 (7)	0.0077 (7)
C554	0.097 (3)	0.076 (5)	0.106 (4)	−0.048 (3)	0.046 (3)	−0.013 (5)
C555	0.144 (6)	0.050 (3)	0.120 (5)	−0.023 (4)	0.071 (7)	0.013 (3)
O51	0.0265 (10)	0.0521 (13)	0.0445 (11)	0.0054 (8)	0.0009 (8)	0.0032 (9)
C51	0.0373 (16)	0.050 (2)	0.0386 (16)	0.0002 (14)	0.0115 (13)	−0.0033 (14)
C52	0.0380 (17)	0.075 (3)	0.0406 (17)	0.0016 (16)	0.0073 (14)	−0.0046 (16)
C53	0.064 (2)	0.100 (4)	0.065 (2)	−0.028 (2)	0.0160 (19)	−0.015 (2)
C54	0.129 (4)	0.073 (4)	0.085 (3)	−0.032 (3)	0.043 (3)	−0.017 (3)
C55	0.137 (4)	0.054 (3)	0.083 (3)	0.018 (3)	0.048 (3)	0.004 (2)
C56	0.069 (2)	0.057 (3)	0.066 (2)	0.0164 (19)	0.0243 (18)	0.0042 (18)
C57	0.0410 (19)	0.119 (4)	0.072 (2)	0.018 (2)	0.0016 (17)	0.001 (2)

### 3'-Methyl-5'-(2-methylphenoxy)-1'-phenyl-5-(thiophen-2-yl)-3,4-dihydro-1'H,2H-3,4'-bipyrazole-2-carbothioamide (Ia) Geometric parameters (Å, º)

**Table:**

N1—C5	1.362 (3)	S421—C422	1.682 (3)
N1—N2	1.379 (2)	C422—N423	1.341 (3)
N1—C11	1.421 (3)	N423—H42A	0.91 (3)
N2—C3	1.326 (3)	N423—H42B	0.90 (3)
C3—C4	1.407 (3)	S451—C455	1.690 (6)
C3—C31	1.491 (3)	S451—C452	1.704 (3)
C4—C5	1.360 (3)	C452—C453	1.331 (8)
C4—C43	1.499 (3)	C453—C454	1.426 (13)
C5—O51	1.366 (3)	C453—H453	0.9300
C11—C12	1.376 (4)	C454—C455	1.343 (7)
C11—C16	1.376 (4)	C454—H454	0.9300
C12—C13	1.389 (4)	C455—H455	0.9300
C12—H12	0.9300	S551—C555	1.690 (12)
C13—C14	1.372 (5)	C553—C554	1.428 (16)
C13—H13	0.9300	C553—H553	0.9300
C14—C15	1.372 (5)	C554—C555	1.344 (12)
C14—H14	0.9300	C554—H554	0.9300
C15—C16	1.382 (4)	C555—H555	0.9300
C15—H15	0.9300	O51—C51	1.406 (3)
C16—H16	0.9300	C51—C56	1.374 (4)
C31—H31A	0.9600	C51—C52	1.382 (4)
C31—H31B	0.9600	C52—C53	1.392 (5)
C31—H31C	0.9600	C52—C57	1.475 (4)
N41—C45	1.280 (3)	C53—C54	1.361 (5)
N41—N42	1.392 (3)	C53—H53	0.9300
N42—C422	1.336 (3)	C54—C55	1.366 (5)
N42—C43	1.487 (3)	C54—H54	0.9300
C43—C44	1.537 (3)	C55—C56	1.387 (4)
C43—H43	0.9800	C55—H55	0.9300
C44—C45	1.501 (3)	C56—H56	0.9300
C44—H44A	0.9700	C57—H57A	0.9600
C44—H44B	0.9700	C57—H57B	0.9600
C45—C452	1.447 (4)	C57—H57C	0.9600
			
C5—N1—N2	109.56 (19)	N41—C45—C44	114.4 (2)
C5—N1—C11	130.2 (2)	C452—C45—C44	124.5 (3)
N2—N1—C11	119.8 (2)	N42—C422—N423	116.4 (2)
C3—N2—N1	105.00 (18)	N42—C422—S421	121.8 (2)
N2—C3—C4	112.4 (2)	N423—C422—S421	121.7 (2)
N2—C3—C31	120.2 (2)	C422—N423—H42A	120.3 (18)
C4—C3—C31	127.4 (2)	C422—N423—H42B	119.2 (18)
C5—C4—C3	103.8 (2)	H42A—N423—H42B	121 (3)
C5—C4—C43	129.7 (2)	C455—S451—C452	91.8 (3)
C3—C4—C43	126.3 (2)	C453—C452—C45	127.5 (7)
C4—C5—N1	109.2 (2)	C453—C452—S451	111.0 (6)
C4—C5—O51	130.8 (2)	C45—C452—S451	121.5 (3)
N1—C5—O51	119.7 (2)	C452—C453—C454	113.8 (8)
C12—C11—C16	120.5 (3)	C452—C453—H453	123.1
C12—C11—N1	120.6 (3)	C454—C453—H453	123.1
C16—C11—N1	118.8 (3)	C455—C454—C453	110.7 (6)
C11—C12—C13	118.8 (3)	C455—C454—H454	124.7
C11—C12—H12	120.6	C453—C454—H454	124.7
C13—C12—H12	120.6	C454—C455—S451	112.7 (5)
C14—C13—C12	120.8 (3)	C454—C455—H455	123.6
C14—C13—H13	119.6	S451—C455—H455	123.6
C12—C13—H13	119.6	C554—C553—H553	123.2
C15—C14—C13	119.8 (3)	C555—C554—C553	110.8 (13)
C15—C14—H14	120.1	C555—C554—H554	124.6
C13—C14—H14	120.1	C553—C554—H554	124.6
C14—C15—C16	120.1 (3)	C554—C555—S551	112.2 (12)
C14—C15—H15	119.9	C554—C555—H555	123.9
C16—C15—H15	119.9	S551—C555—H555	123.9
C11—C16—C15	119.9 (3)	C5—O51—C51	117.9 (2)
C11—C16—H16	120.0	C56—C51—C52	122.6 (3)
C15—C16—H16	120.0	C56—C51—O51	122.3 (2)
C3—C31—H31A	109.5	C52—C51—O51	115.1 (3)
C3—C31—H31B	109.5	C51—C52—C53	116.4 (3)
H31A—C31—H31B	109.5	C51—C52—C57	121.8 (3)
C3—C31—H31C	109.5	C53—C52—C57	121.7 (3)
H31A—C31—H31C	109.5	C54—C53—C52	121.7 (4)
H31B—C31—H31C	109.5	C54—C53—H53	119.2
C45—N41—N42	108.1 (2)	C52—C53—H53	119.2
C422—N42—N41	119.9 (2)	C53—C54—C55	121.0 (4)
C422—N42—C43	127.2 (2)	C53—C54—H54	119.5
N41—N42—C43	112.9 (2)	C55—C54—H54	119.5
N42—C43—C4	112.3 (2)	C54—C55—C56	119.2 (4)
N42—C43—C44	101.01 (19)	C54—C55—H55	120.4
C4—C43—C44	116.4 (2)	C56—C55—H55	120.4
N42—C43—H43	108.9	C51—C56—C55	119.2 (3)
C4—C43—H43	108.9	C51—C56—H56	120.4
C44—C43—H43	108.9	C55—C56—H56	120.4
C45—C44—C43	102.9 (2)	C52—C57—H57A	109.5
C45—C44—H44A	111.2	C52—C57—H57B	109.5
C43—C44—H44A	111.2	H57A—C57—H57B	109.5
C45—C44—H44B	111.2	C52—C57—H57C	109.5
C43—C44—H44B	111.2	H57A—C57—H57C	109.5
H44A—C44—H44B	109.1	H57B—C57—H57C	109.5
N41—C45—C452	121.1 (3)		
			
C5—N1—N2—C3	−0.7 (3)	N42—C43—C44—C45	−7.5 (2)
C11—N1—N2—C3	−173.9 (2)	C4—C43—C44—C45	114.3 (2)
N1—N2—C3—C4	1.1 (3)	N42—N41—C45—C452	179.1 (2)
N1—N2—C3—C31	−178.5 (2)	N42—N41—C45—C44	−1.3 (3)
N2—C3—C4—C5	−1.1 (3)	C43—C44—C45—N41	6.1 (3)
C31—C3—C4—C5	178.5 (3)	C43—C44—C45—C452	−174.4 (2)
N2—C3—C4—C43	174.5 (3)	N41—N42—C422—N423	4.6 (3)
C31—C3—C4—C43	−5.8 (5)	C43—N42—C422—N423	−177.0 (2)
C3—C4—C5—N1	0.6 (3)	N41—N42—C422—S421	−174.18 (16)
C43—C4—C5—N1	−174.8 (3)	C43—N42—C422—S421	4.2 (3)
C3—C4—C5—O51	173.7 (3)	N41—C45—C452—C453	−178.0 (11)
C43—C4—C5—O51	−1.7 (5)	C44—C45—C452—C453	2.5 (12)
N2—N1—C5—C4	0.0 (3)	N41—C45—C452—S451	0.3 (4)
C11—N1—C5—C4	172.3 (3)	C44—C45—C452—S451	−179.2 (2)
N2—N1—C5—O51	−174.0 (2)	C455—S451—C452—C453	1.4 (10)
C11—N1—C5—O51	−1.7 (4)	C455—S451—C452—C45	−177.2 (4)
C5—N1—C11—C12	45.3 (4)	C45—C452—C453—C454	177.6 (11)
N2—N1—C11—C12	−143.1 (3)	S451—C452—C453—C454	−0.9 (18)
C5—N1—C11—C16	−135.0 (3)	C452—C453—C454—C455	0 (2)
N2—N1—C11—C16	36.6 (4)	C453—C454—C455—S451	1.4 (18)
C16—C11—C12—C13	2.0 (5)	C452—S451—C455—C454	−1.6 (11)
N1—C11—C12—C13	−178.3 (3)	C553—C554—C555—S551	−4 (13)
C11—C12—C13—C14	−1.6 (5)	C4—C5—O51—C51	76.6 (4)
C12—C13—C14—C15	−0.1 (6)	N1—C5—O51—C51	−110.9 (3)
C13—C14—C15—C16	1.3 (5)	C5—O51—C51—C56	22.5 (3)
C12—C11—C16—C15	−0.8 (4)	C5—O51—C51—C52	−158.9 (2)
N1—C11—C16—C15	179.5 (3)	C56—C51—C52—C53	0.2 (4)
C14—C15—C16—C11	−0.9 (5)	O51—C51—C52—C53	−178.3 (2)
C45—N41—N42—C422	174.3 (2)	C56—C51—C52—C57	−178.4 (3)
C45—N41—N42—C43	−4.4 (3)	O51—C51—C52—C57	3.1 (4)
C422—N42—C43—C4	64.4 (3)	C51—C52—C53—C54	−0.2 (5)
N41—N42—C43—C4	−117.0 (2)	C57—C52—C53—C54	178.4 (3)
C422—N42—C43—C44	−170.8 (2)	C52—C53—C54—C55	−0.5 (6)
N41—N42—C43—C44	7.7 (3)	C53—C54—C55—C56	1.1 (6)
C5—C4—C43—N42	41.7 (4)	C52—C51—C56—C55	0.4 (4)
C3—C4—C43—N42	−132.8 (3)	O51—C51—C56—C55	178.8 (2)
C5—C4—C43—C44	−74.0 (4)	C54—C55—C56—C51	−1.0 (5)
C3—C4—C43—C44	111.5 (3)		

### 3'-Methyl-5'-(2-methylphenoxy)-1'-phenyl-5-(thiophen-2-yl)-3,4-dihydro-1'H,2H-3,4'-bipyrazole-2-carbothioamide (Ia) Hydrogen-bond geometry (Å, º)

**Table:**

*D*—H···*A*	*D*—H	H···*A*	*D*···*A*	*D*—H···*A*
N423—H42*A*···N2^i^	0.92 (3)	2.28 (3)	3.111 (3)	150 (2)
N423—H42*A*···N41	0.92 (3)	2.27 (3)	2.628 (4)	103 (2)

Symmetry code: (i) *x*+1, *y*, *z*.

### 5'-(2,4-Dichlorophenoxy)-3'-methyl-1'-phenyl-5-(thiophen-2-yl)-3,4-dihydro-1'H,2H-3,4'-bipyrazole-2-carbothioamide hemihydrate (Ib) Crystal data

**Table:**

2C_24_H_19_Cl_2_N_5_OS_2_·H_2_O	*F*(000) = 1108
*M**_r_* = 1074.94	*D*_x_ = 1.456 Mg m^−^^3^
Monoclinic, *P*2/*c*	Mo *K*α radiation, λ = 0.71073 Å
*a* = 15.037 (1) Å	Cell parameters from 5307 reflections
*b* = 8.4266 (6) Å	θ = 2.6–27.9°
*c* = 19.471 (1) Å	µ = 0.47 mm^−^^1^
β = 96.246 (6)°	*T* = 296 K
*V* = 2452.5 (3) Å^3^	Needle, orange
*Z* = 2	0.36 × 0.12 × 0.12 mm

### 5'-(2,4-Dichlorophenoxy)-3'-methyl-1'-phenyl-5-(thiophen-2-yl)-3,4-dihydro-1'H,2H-3,4'-bipyrazole-2-carbothioamide hemihydrate (Ib) Data collection

**Table:**

Oxford Diffraction Xcalibur with Sapphire CCD detector diffractometer	5290 independent reflections
Radiation source: Enhance (Mo) X-ray Source	3075 reflections with *I* > 2σ(*I*)
Graphite monochromator	*R*_int_ = 0.033
ω scans	θ_max_ = 27.6°, θ_min_ = 2.6°
Absorption correction: multi-scan (CrysAlis RED; Oxford Diffraction, 2009)	*h* = −19→13
*T*_min_ = 0.922, *T*_max_ = 0.946	*k* = −10→10
10494 measured reflections	*l* = −25→25

### 5'-(2,4-Dichlorophenoxy)-3'-methyl-1'-phenyl-5-(thiophen-2-yl)-3,4-dihydro-1'H,2H-3,4'-bipyrazole-2-carbothioamide hemihydrate (Ib) Refinement

**Table:**

Refinement on *F*^2^	Primary atom site location: difference Fourier map
Least-squares matrix: full	Hydrogen site location: mixed
*R*[*F*^2^ > 2σ(*F*^2^)] = 0.046	H atoms treated by a mixture of independent and constrained refinement
*wR*(*F*^2^) = 0.097	*w* = 1/[σ^2^(*F*_o_^2^) + (0.0411*P*)^2^] where *P* = (*F*_o_^2^ + 2*F*_c_^2^)/3
*S* = 0.98	(Δ/σ)_max_ = 0.001
5290 reflections	Δρ_max_ = 0.26 e Å^−^^3^
335 parameters	Δρ_min_ = −0.30 e Å^−^^3^
10 restraints	

### 5'-(2,4-Dichlorophenoxy)-3'-methyl-1'-phenyl-5-(thiophen-2-yl)-3,4-dihydro-1'H,2H-3,4'-bipyrazole-2-carbothioamide hemihydrate (Ib) Special details

**Table:**

Experimental. CrysAlis RED, Oxford Diffraction Ltd., 2009 Empirical absorption correction using spherical harmonics, implemented in SCALE3 ABSPACK scaling algorithm.
Geometry. All esds (except the esd in the dihedral angle between two l.s. planes) are estimated using the full covariance matrix. The cell esds are taken into account individually in the estimation of esds in distances, angles and torsion angles; correlations between esds in cell parameters are only used when they are defined by crystal symmetry. An approximate (isotropic) treatment of cell esds is used for estimating esds involving l.s. planes.

### 5'-(2,4-Dichlorophenoxy)-3'-methyl-1'-phenyl-5-(thiophen-2-yl)-3,4-dihydro-1'H,2H-3,4'-bipyrazole-2-carbothioamide hemihydrate (Ib) Fractional atomic coordinates and isotropic or equivalent isotropic displacement parameters (Å^2^)

**Table:**

	*x*	*y*	*z*	*U*_iso_*/*U*_eq_	Occ. (<1)
N1	0.68939 (13)	0.4363 (2)	0.37947 (9)	0.0343 (5)	
N2	0.61354 (13)	0.3752 (2)	0.34400 (9)	0.0356 (5)	
C3	0.59656 (15)	0.2419 (3)	0.37692 (11)	0.0318 (5)	
C4	0.66061 (15)	0.2149 (3)	0.43422 (11)	0.0296 (5)	
C5	0.71767 (15)	0.3404 (3)	0.43322 (11)	0.0308 (5)	
C11	0.72930 (16)	0.5768 (3)	0.35564 (11)	0.0354 (6)	
C12	0.81789 (18)	0.5775 (3)	0.34460 (15)	0.0602 (8)	
H12	0.8535	0.4889	0.3557	0.072*	
C13	0.8538 (2)	0.7111 (4)	0.31678 (17)	0.0748 (10)	
H13	0.9137	0.7127	0.3089	0.090*	
C14	0.8006 (2)	0.8415 (3)	0.30086 (14)	0.0580 (8)	
H14	0.8244	0.9307	0.2815	0.070*	
C15	0.7138 (2)	0.8410 (3)	0.31316 (13)	0.0471 (7)	
H15	0.6788	0.9306	0.3028	0.057*	
C16	0.67667 (17)	0.7089 (3)	0.34090 (11)	0.0384 (6)	
H16	0.6170	0.7092	0.3495	0.046*	
C31	0.51811 (17)	0.1418 (3)	0.35213 (13)	0.0438 (6)	
H31A	0.4846	0.1180	0.3901	0.066*	
H31B	0.5384	0.0447	0.3332	0.066*	
H31C	0.4807	0.1979	0.3171	0.066*	
N41	0.75120 (13)	0.0972 (2)	0.59603 (9)	0.0355 (5)	
N42	0.67049 (13)	0.1362 (2)	0.55799 (9)	0.0344 (5)	
C43	0.66437 (16)	0.0806 (3)	0.48505 (11)	0.0335 (6)	
H43	0.6115	0.0131	0.4751	0.040*	
C44	0.74925 (17)	−0.0209 (3)	0.48690 (12)	0.0404 (6)	
H44A	0.7346	−0.1326	0.4813	0.048*	
H44B	0.7855	0.0110	0.4510	0.048*	
C45	0.79663 (16)	0.0117 (3)	0.55736 (11)	0.0336 (6)	
S421	0.50684 (4)	0.25555 (8)	0.54387 (3)	0.0477 (2)	
C422	0.60539 (16)	0.2113 (3)	0.58829 (12)	0.0361 (6)	
N423	0.62484 (18)	0.2475 (3)	0.65504 (11)	0.0512 (7)	
H42A	0.585 (2)	0.293 (3)	0.6745 (14)	0.061*	
H42B	0.675 (2)	0.220 (3)	0.6753 (14)	0.061*	
S451	0.93613 (5)	−0.02351 (9)	0.66256 (4)	0.0493 (3)	0.951 (3)
C452	0.88401 (16)	−0.0510 (3)	0.58006 (12)	0.0365 (6)	0.951 (3)
C453	0.9356 (3)	−0.1417 (6)	0.5435 (2)	0.0475 (9)	0.951 (3)
H453	0.9188	−0.1700	0.4977	0.057*	0.951 (3)
C454	1.0167 (2)	−0.1900 (7)	0.5800 (2)	0.0524 (14)	0.951 (3)
H454	1.0593	−0.2517	0.5612	0.063*	0.951 (3)
C455	1.0251 (2)	−0.1363 (4)	0.64507 (18)	0.0537 (12)	0.951 (3)
H455	1.0740	−0.1580	0.6772	0.064*	0.951 (3)
S551	0.937 (2)	−0.159 (4)	0.5215 (11)	0.0475 (9)	0.049 (3)
C552	0.88401 (16)	−0.0510 (3)	0.58006 (12)	0.0365 (6)	0.049 (3)
C553	0.944 (2)	−0.005 (7)	0.633 (2)	0.0493 (3)	0.049 (3)
H553	0.9341	0.0767	0.6631	0.059*	0.049 (3)
C554	1.024 (3)	−0.094 (11)	0.637 (3)	0.0537 (12)	0.049 (3)
H554	1.0649	−0.1006	0.6763	0.064*	0.049 (3)
C555	1.032 (4)	−0.169 (16)	0.577 (4)	0.0524 (14)	0.049 (3)
H555	1.0839	−0.2211	0.5676	0.063*	0.049 (3)
O51	0.79155 (10)	0.37875 (17)	0.47791 (7)	0.0338 (4)	
C51	0.77558 (16)	0.4711 (3)	0.53498 (11)	0.0326 (6)	
C52	0.84468 (16)	0.4851 (3)	0.58742 (12)	0.0366 (6)	
Cl52	0.94533 (5)	0.39239 (9)	0.58023 (4)	0.0618 (2)	
C53	0.83348 (19)	0.5733 (3)	0.64567 (13)	0.0464 (7)	
H53	0.8799	0.5827	0.6811	0.056*	
C54	0.7533 (2)	0.6467 (3)	0.65044 (13)	0.0453 (7)	
Cl54	0.73886 (6)	0.75872 (9)	0.72375 (4)	0.0733 (3)	
C55	0.68368 (19)	0.6329 (3)	0.59891 (14)	0.0496 (7)	
H55	0.6294	0.6826	0.6032	0.060*	
C56	0.69494 (17)	0.5446 (3)	0.54082 (13)	0.0424 (6)	
H56	0.6482	0.5348	0.5057	0.051*	
O61	0.5000	0.5746 (3)	0.2500	0.0605 (9)	
H61	0.532 (2)	0.511 (3)	0.2784 (15)	0.091*	

### 5'-(2,4-Dichlorophenoxy)-3'-methyl-1'-phenyl-5-(thiophen-2-yl)-3,4-dihydro-1'H,2H-3,4'-bipyrazole-2-carbothioamide hemihydrate (Ib) Atomic displacement parameters (Å^2^)

**Table:**

	*U*^11^	*U*^22^	*U*^33^	*U*^12^	*U*^13^	*U*^23^
N1	0.0301 (11)	0.0402 (12)	0.0312 (11)	−0.0022 (9)	−0.0028 (9)	0.0066 (9)
N2	0.0304 (12)	0.0441 (12)	0.0306 (11)	−0.0018 (10)	−0.0039 (9)	0.0035 (9)
C3	0.0300 (13)	0.0390 (14)	0.0263 (12)	0.0011 (11)	0.0029 (10)	−0.0019 (11)
C4	0.0306 (13)	0.0325 (13)	0.0254 (12)	0.0022 (11)	0.0013 (10)	0.0006 (10)
C5	0.0259 (13)	0.0372 (14)	0.0280 (12)	0.0040 (11)	−0.0024 (10)	−0.0010 (11)
C11	0.0334 (14)	0.0411 (15)	0.0309 (13)	−0.0047 (12)	0.0001 (11)	0.0056 (11)
C12	0.0372 (17)	0.0615 (19)	0.082 (2)	0.0038 (15)	0.0074 (16)	0.0298 (17)
C13	0.0392 (18)	0.087 (2)	0.098 (3)	−0.0103 (18)	0.0100 (18)	0.039 (2)
C14	0.058 (2)	0.0583 (19)	0.0562 (18)	−0.0216 (17)	−0.0028 (16)	0.0207 (15)
C15	0.0563 (19)	0.0407 (16)	0.0432 (15)	−0.0018 (14)	0.0001 (14)	0.0049 (13)
C16	0.0383 (15)	0.0438 (15)	0.0332 (14)	−0.0012 (12)	0.0046 (12)	−0.0012 (12)
C31	0.0385 (15)	0.0491 (16)	0.0421 (15)	−0.0018 (13)	−0.0030 (12)	−0.0019 (12)
N41	0.0348 (12)	0.0398 (12)	0.0314 (11)	0.0032 (10)	0.0015 (9)	0.0036 (9)
N42	0.0341 (12)	0.0424 (12)	0.0264 (10)	0.0080 (10)	0.0017 (9)	0.0004 (9)
C43	0.0367 (14)	0.0345 (13)	0.0286 (12)	−0.0024 (11)	0.0009 (11)	−0.0001 (11)
C44	0.0496 (16)	0.0359 (14)	0.0348 (13)	0.0073 (13)	0.0010 (12)	−0.0001 (11)
C45	0.0412 (15)	0.0284 (13)	0.0313 (13)	0.0013 (12)	0.0047 (12)	0.0032 (11)
S421	0.0361 (4)	0.0614 (5)	0.0457 (4)	0.0054 (3)	0.0045 (3)	0.0036 (3)
C422	0.0382 (15)	0.0369 (14)	0.0336 (13)	−0.0021 (12)	0.0062 (12)	0.0045 (11)
N423	0.0508 (16)	0.0714 (17)	0.0317 (13)	0.0165 (13)	0.0051 (11)	−0.0039 (12)
S451	0.0460 (5)	0.0588 (5)	0.0412 (5)	0.0101 (4)	−0.0039 (4)	−0.0069 (4)
C452	0.0382 (15)	0.0347 (14)	0.0361 (13)	0.0032 (12)	0.0026 (12)	0.0038 (11)
C453	0.0523 (18)	0.057 (2)	0.035 (3)	0.0135 (15)	0.009 (2)	0.000 (2)
C454	0.041 (2)	0.058 (3)	0.0590 (19)	0.013 (2)	0.0074 (17)	−0.0020 (17)
C455	0.0405 (17)	0.056 (3)	0.061 (2)	0.0099 (16)	−0.0100 (15)	0.0011 (18)
S551	0.0523 (18)	0.057 (2)	0.035 (3)	0.0135 (15)	0.009 (2)	0.000 (2)
C552	0.0382 (15)	0.0347 (14)	0.0361 (13)	0.0032 (12)	0.0026 (12)	0.0038 (11)
C553	0.0460 (5)	0.0588 (5)	0.0412 (5)	0.0101 (4)	−0.0039 (4)	−0.0069 (4)
C554	0.0405 (17)	0.056 (3)	0.061 (2)	0.0099 (16)	−0.0100 (15)	0.0011 (18)
C555	0.041 (2)	0.058 (3)	0.0590 (19)	0.013 (2)	0.0074 (17)	−0.0020 (17)
O51	0.0261 (9)	0.0424 (10)	0.0317 (9)	0.0033 (7)	−0.0025 (7)	−0.0042 (8)
C51	0.0356 (14)	0.0287 (13)	0.0333 (13)	−0.0006 (11)	0.0031 (11)	0.0024 (11)
C52	0.0320 (14)	0.0378 (14)	0.0392 (14)	0.0002 (11)	−0.0004 (12)	0.0004 (12)
Cl52	0.0389 (4)	0.0757 (5)	0.0665 (5)	0.0146 (4)	−0.0133 (4)	−0.0190 (4)
C53	0.0496 (18)	0.0501 (17)	0.0385 (15)	−0.0095 (14)	0.0007 (13)	−0.0065 (13)
C54	0.0579 (19)	0.0391 (15)	0.0411 (15)	−0.0095 (14)	0.0155 (14)	−0.0061 (12)
Cl54	0.0881 (6)	0.0778 (6)	0.0589 (5)	−0.0104 (5)	0.0293 (4)	−0.0278 (4)
C55	0.0437 (17)	0.0510 (17)	0.0564 (18)	0.0062 (13)	0.0157 (15)	−0.0078 (14)
C56	0.0352 (15)	0.0455 (16)	0.0454 (15)	0.0029 (12)	−0.0004 (13)	−0.0032 (13)
O61	0.064 (2)	0.0498 (18)	0.0594 (19)	0.000	−0.0293 (15)	0.000

### 5'-(2,4-Dichlorophenoxy)-3'-methyl-1'-phenyl-5-(thiophen-2-yl)-3,4-dihydro-1'H,2H-3,4'-bipyrazole-2-carbothioamide hemihydrate (Ib) Geometric parameters (Å, º)

**Table:**

N1—C5	1.354 (3)	C45—C452	1.440 (3)
N1—N2	1.368 (2)	S421—C422	1.675 (2)
N1—C11	1.427 (3)	C422—N423	1.336 (3)
N2—C3	1.332 (3)	N423—H42A	0.83 (3)
C3—C4	1.411 (3)	N423—H42B	0.84 (3)
C3—C31	1.488 (3)	S451—C455	1.705 (3)
C4—C5	1.363 (3)	S451—C452	1.725 (2)
C4—C43	1.500 (3)	C452—C453	1.347 (5)
C5—O51	1.373 (2)	C453—C454	1.404 (5)
C11—C12	1.372 (3)	C453—H453	0.9300
C11—C16	1.378 (3)	C454—C455	1.339 (4)
C12—C13	1.385 (4)	C454—H454	0.9300
C12—H12	0.9300	C455—H455	0.9300
C13—C14	1.375 (4)	S551—C555	1.707 (11)
C13—H13	0.9300	C553—C554	1.407 (11)
C14—C15	1.352 (4)	C553—H553	0.9300
C14—H14	0.9300	C554—C555	1.342 (10)
C15—C16	1.382 (3)	C554—H554	0.9300
C15—H15	0.9300	C555—H555	0.9300
C16—H16	0.9300	O51—C51	1.399 (3)
C31—H31A	0.9600	C51—C56	1.377 (3)
C31—H31B	0.9600	C51—C52	1.380 (3)
C31—H31C	0.9600	C52—C53	1.382 (3)
N41—C45	1.290 (3)	C52—Cl52	1.722 (2)
N41—N42	1.391 (2)	C53—C54	1.366 (4)
N42—C422	1.354 (3)	C53—H53	0.9300
N42—C43	1.489 (3)	C54—C55	1.374 (4)
C43—C44	1.534 (3)	C54—Cl54	1.745 (3)
C43—H43	0.9800	C55—C56	1.379 (3)
C44—C45	1.501 (3)	C55—H55	0.9300
C44—H44A	0.9700	C56—H56	0.9300
C44—H44B	0.9700	O61—H61	0.88 (3)
			
C5—N1—N2	109.89 (18)	C43—C44—H44B	111.1
C5—N1—C11	129.69 (19)	H44A—C44—H44B	109.1
N2—N1—C11	120.32 (17)	N41—C45—C452	123.3 (2)
C3—N2—N1	105.50 (17)	N41—C45—C44	114.0 (2)
N2—C3—C4	111.5 (2)	C452—C45—C44	122.7 (2)
N2—C3—C31	120.6 (2)	N423—C422—N42	116.0 (2)
C4—C3—C31	127.9 (2)	N423—C422—S421	122.6 (2)
C5—C4—C3	103.90 (19)	N42—C422—S421	121.36 (18)
C5—C4—C43	128.0 (2)	C422—N423—H42A	117.2 (19)
C3—C4—C43	128.1 (2)	C422—N423—H42B	118.9 (19)
N1—C5—C4	109.20 (19)	H42A—N423—H42B	124 (3)
N1—C5—O51	120.9 (2)	C455—S451—C452	91.49 (14)
C4—C5—O51	129.8 (2)	C453—C452—C45	127.2 (3)
C12—C11—C16	120.6 (2)	C453—C452—S451	109.8 (2)
C12—C11—N1	120.3 (2)	C45—C452—S451	122.94 (19)
C16—C11—N1	119.1 (2)	C452—C453—C454	114.6 (3)
C11—C12—C13	119.5 (3)	C452—C453—H453	122.7
C11—C12—H12	120.3	C454—C453—H453	122.7
C13—C12—H12	120.3	C455—C454—C453	111.6 (3)
C14—C13—C12	119.7 (3)	C455—C454—H454	124.2
C14—C13—H13	120.1	C453—C454—H454	124.2
C12—C13—H13	120.1	C454—C455—S451	112.5 (2)
C15—C14—C13	120.5 (3)	C454—C455—H455	123.7
C15—C14—H14	119.8	S451—C455—H455	123.7
C13—C14—H14	119.8	C554—C553—H553	123.6
C14—C15—C16	120.7 (3)	C555—C554—C553	111.1 (15)
C14—C15—H15	119.6	C555—C554—H554	124.5
C16—C15—H15	119.6	C553—C554—H554	124.5
C11—C16—C15	119.0 (2)	C554—C555—S551	112.1 (11)
C11—C16—H16	120.5	C554—C555—H555	123.9
C15—C16—H16	120.5	S551—C555—H555	123.9
C3—C31—H31A	109.5	C5—O51—C51	115.94 (17)
C3—C31—H31B	109.5	C56—C51—C52	120.0 (2)
H31A—C31—H31B	109.5	C56—C51—O51	123.0 (2)
C3—C31—H31C	109.5	C52—C51—O51	117.0 (2)
H31A—C31—H31C	109.5	C51—C52—C53	120.2 (2)
H31B—C31—H31C	109.5	C51—C52—Cl52	119.92 (18)
C45—N41—N42	107.92 (18)	C53—C52—Cl52	119.83 (19)
C422—N42—N41	120.58 (18)	C54—C53—C52	119.1 (2)
C422—N42—C43	126.17 (19)	C54—C53—H53	120.4
N41—N42—C43	113.19 (18)	C52—C53—H53	120.4
N42—C43—C4	112.72 (18)	C53—C54—C55	121.3 (2)
N42—C43—C44	100.84 (17)	C53—C54—Cl54	119.3 (2)
C4—C43—C44	114.06 (19)	C55—C54—Cl54	119.3 (2)
N42—C43—H43	109.6	C54—C55—C56	119.5 (2)
C4—C43—H43	109.6	C54—C55—H55	120.2
C44—C43—H43	109.6	C56—C55—H55	120.2
C45—C44—C43	103.32 (18)	C51—C56—C55	119.9 (2)
C45—C44—H44A	111.1	C51—C56—H56	120.1
C43—C44—H44A	111.1	C55—C56—H56	120.1
C45—C44—H44B	111.1		
			
C5—N1—N2—C3	0.0 (2)	C4—C43—C44—C45	113.3 (2)
C11—N1—N2—C3	176.76 (19)	N42—N41—C45—C452	−179.7 (2)
N1—N2—C3—C4	0.3 (2)	N42—N41—C45—C44	−1.3 (3)
N1—N2—C3—C31	−179.6 (2)	C43—C44—C45—N41	6.2 (3)
N2—C3—C4—C5	−0.5 (2)	C43—C44—C45—C452	−175.4 (2)
C31—C3—C4—C5	179.4 (2)	N41—N42—C422—N423	1.5 (3)
N2—C3—C4—C43	178.8 (2)	C43—N42—C422—N423	178.6 (2)
C31—C3—C4—C43	−1.4 (4)	N41—N42—C422—S421	−178.60 (16)
N2—N1—C5—C4	−0.3 (3)	C43—N42—C422—S421	−1.5 (3)
C11—N1—C5—C4	−176.7 (2)	N41—C45—C452—C453	179.5 (4)
N2—N1—C5—O51	−178.18 (18)	C44—C45—C452—C453	1.3 (5)
C11—N1—C5—O51	5.4 (3)	N41—C45—C452—S451	2.1 (3)
C3—C4—C5—N1	0.4 (2)	C44—C45—C452—S451	−176.17 (18)
C43—C4—C5—N1	−178.8 (2)	C455—S451—C452—C453	−0.4 (3)
C3—C4—C5—O51	178.1 (2)	C455—S451—C452—C45	177.4 (2)
C43—C4—C5—O51	−1.1 (4)	C45—C452—C453—C454	−177.9 (4)
C5—N1—C11—C12	50.0 (3)	S451—C452—C453—C454	−0.2 (6)
N2—N1—C11—C12	−126.0 (3)	C452—C453—C454—C455	0.9 (7)
C5—N1—C11—C16	−132.9 (2)	C453—C454—C455—S451	−1.2 (6)
N2—N1—C11—C16	51.0 (3)	C452—S451—C455—C454	0.9 (4)
C16—C11—C12—C13	−1.6 (4)	C553—C554—C555—S551	−10 (12)
N1—C11—C12—C13	175.4 (2)	N1—C5—O51—C51	89.3 (2)
C11—C12—C13—C14	0.3 (5)	C4—C5—O51—C51	−88.1 (3)
C12—C13—C14—C15	1.0 (5)	C5—O51—C51—C56	−11.5 (3)
C13—C14—C15—C16	−1.0 (4)	C5—O51—C51—C52	167.5 (2)
C12—C11—C16—C15	1.6 (4)	C56—C51—C52—C53	−0.4 (3)
N1—C11—C16—C15	−175.40 (19)	O51—C51—C52—C53	−179.4 (2)
C14—C15—C16—C11	−0.3 (4)	C56—C51—C52—Cl52	179.72 (18)
C45—N41—N42—C422	172.8 (2)	O51—C51—C52—Cl52	0.7 (3)
C45—N41—N42—C43	−4.6 (2)	C51—C52—C53—C54	−0.2 (4)
C422—N42—C43—C4	68.7 (3)	Cl52—C52—C53—C54	179.7 (2)
N41—N42—C43—C4	−114.0 (2)	C52—C53—C54—C55	0.7 (4)
C422—N42—C43—C44	−169.3 (2)	C52—C53—C54—Cl54	−179.85 (18)
N41—N42—C43—C44	8.0 (2)	C53—C54—C55—C56	−0.7 (4)
C5—C4—C43—N42	52.6 (3)	Cl54—C54—C55—C56	179.89 (19)
C3—C4—C43—N42	−126.5 (2)	C52—C51—C56—C55	0.4 (4)
C5—C4—C43—C44	−61.7 (3)	O51—C51—C56—C55	179.4 (2)
C3—C4—C43—C44	119.3 (3)	C54—C55—C56—C51	0.1 (4)
N42—C43—C44—C45	−7.8 (2)		

### 5'-(2,4-Dichlorophenoxy)-3'-methyl-1'-phenyl-5-(thiophen-2-yl)-3,4-dihydro-1'H,2H-3,4'-bipyrazole-2-carbothioamide hemihydrate (Ib) Hydrogen-bond geometry (Å, º)

**Table:**

*D*—H···*A*	*D*—H	H···*A*	*D*···*A*	*D*—H···*A*
O61—H61···N2	0.88 (3)	2.03 (3)	2.900 (2)	176 (2)
N423—H42*A*···O61^i^	0.84 (3)	2.33 (3)	3.154 (3)	167 (3)
N423—H42*B*···N41	0.85 (3)	2.27 (3)	2.648 (3)	107 (2)

Symmetry code: (i) −*x*+1, −*y*+1, −*z*+1.

### Ethyl (Z)-2-{2-[3'-methyl-1'-phenyl-5-(thiophen-2-yl)-5'-(2-methylphenoxy)-3,4-dihydro-1'H,2H-3,4'-bipyrazole-2-yl]-4-oxo-4,5-dihydrothiazol-5-ylidene}acetate (II) Crystal data

**Table:**

C_31_H_27_N_5_O_4_S_2_	*Z* = 2
*M**_r_* = 597.69	*F*(000) = 624
Triclinic, *P*1	*D*_x_ = 1.331 Mg m^−^^3^
*a* = 10.783 (2) Å	Mo *K*α radiation, λ = 0.71073 Å
*b* = 11.683 (3) Å	Cell parameters from 6462 reflections
*c* = 13.577 (3) Å	θ = 2.5–28.2°
α = 93.54 (2)°	µ = 0.22 mm^−^^1^
β = 105.17 (2)°	*T* = 296 K
γ = 113.20 (2)°	Needle, yellow
*V* = 1490.9 (6) Å^3^	0.48 × 0.12 × 0.06 mm

### Ethyl (Z)-2-{2-[3'-methyl-1'-phenyl-5-(thiophen-2-yl)-5'-(2-methylphenoxy)-3,4-dihydro-1'H,2H-3,4'-bipyrazole-2-yl]-4-oxo-4,5-dihydrothiazol-5-ylidene}acetate (II) Data collection

**Table:**

Oxford Diffraction Xcalibur with Sapphire CCD detector diffractometer	5561 independent reflections
Radiation source: Enhance (Mo) X-ray Source	1730 reflections with *I* > 2σ(*I*)
Graphite monochromator	*R*_int_ = 0.138
ω scans	θ_max_ = 25.6°, θ_min_ = 2.5°
Absorption correction: multi-scan (CrysAlis RED; Oxford Diffraction, 2009)	*h* = −7→13
*T*_min_ = 0.849, *T*_max_ = 0.987	*k* = −14→12
10433 measured reflections	*l* = −16→16

### Ethyl (Z)-2-{2-[3'-methyl-1'-phenyl-5-(thiophen-2-yl)-5'-(2-methylphenoxy)-3,4-dihydro-1'H,2H-3,4'-bipyrazole-2-yl]-4-oxo-4,5-dihydrothiazol-5-ylidene}acetate (II) Refinement

**Table:**

Refinement on *F*^2^	Primary atom site location: difference Fourier map
Least-squares matrix: full	Hydrogen site location: inferred from neighbouring sites
*R*[*F*^2^ > 2σ(*F*^2^)] = 0.079	H-atom parameters constrained
*wR*(*F*^2^) = 0.143	*w* = 1/[σ^2^(*F*_o_^2^) + (0.0319*P*)^2^] where *P* = (*F*_o_^2^ + 2*F*_c_^2^)/3
*S* = 0.88	(Δ/σ)_max_ < 0.001
5561 reflections	Δρ_max_ = 0.26 e Å^−^^3^
395 parameters	Δρ_min_ = −0.24 e Å^−^^3^
10 restraints	

### Ethyl (Z)-2-{2-[3'-methyl-1'-phenyl-5-(thiophen-2-yl)-5'-(2-methylphenoxy)-3,4-dihydro-1'H,2H-3,4'-bipyrazole-2-yl]-4-oxo-4,5-dihydrothiazol-5-ylidene}acetate (II) Special details

**Table:**

Experimental. CrysAlis RED, Oxford Diffraction Ltd., 2009 Empirical absorption correction using spherical harmonics, implemented in SCALE3 ABSPACK scaling algorithm.
Geometry. All esds (except the esd in the dihedral angle between two l.s. planes) are estimated using the full covariance matrix. The cell esds are taken into account individually in the estimation of esds in distances, angles and torsion angles; correlations between esds in cell parameters are only used when they are defined by crystal symmetry. An approximate (isotropic) treatment of cell esds is used for estimating esds involving l.s. planes.

### Ethyl (Z)-2-{2-[3'-methyl-1'-phenyl-5-(thiophen-2-yl)-5'-(2-methylphenoxy)-3,4-dihydro-1'H,2H-3,4'-bipyrazole-2-yl]-4-oxo-4,5-dihydrothiazol-5-ylidene}acetate (II) Fractional atomic coordinates and isotropic or equivalent isotropic displacement parameters (Å^2^)

**Table:**

	*x*	*y*	*z*	*U*_iso_*/*U*_eq_	Occ. (<1)
N1	1.0895 (5)	0.0030 (4)	0.1894 (3)	0.0436 (12)	
N2	1.1527 (4)	0.1065 (4)	0.1476 (4)	0.0459 (12)	
C3	1.0468 (6)	0.1321 (4)	0.0945 (4)	0.0430 (15)	
C4	0.9141 (5)	0.0459 (5)	0.0992 (4)	0.0401 (14)	
C5	0.9473 (6)	−0.0321 (5)	0.1612 (4)	0.0424 (15)	
C11	1.1772 (6)	−0.0460 (5)	0.2534 (4)	0.0420 (14)	
C12	1.1198 (6)	−0.1658 (5)	0.2763 (5)	0.0571 (17)	
H12	1.0229	−0.2171	0.2490	0.068*	
C13	1.2090 (7)	−0.2083 (5)	0.3410 (5)	0.0644 (18)	
H13	1.1707	−0.2886	0.3570	0.077*	
C14	1.3536 (7)	−0.1337 (6)	0.3821 (5)	0.0657 (18)	
H14	1.4123	−0.1629	0.4256	0.079*	
C15	1.4082 (6)	−0.0162 (6)	0.3573 (5)	0.0596 (17)	
H15	1.5054	0.0343	0.3832	0.071*	
C16	1.3214 (6)	0.0286 (5)	0.2947 (4)	0.0459 (14)	
H16	1.3602	0.1096	0.2801	0.055*	
C31	1.0776 (5)	0.2433 (4)	0.0406 (4)	0.0609 (17)	
H31A	1.0234	0.2153	−0.0317	0.091*	
H31B	1.1770	0.2823	0.0477	0.091*	
H31C	1.0520	0.3037	0.0711	0.091*	
N41	0.5871 (4)	−0.0182 (4)	0.1331 (3)	0.0455 (12)	
N42	0.7101 (4)	0.0797 (4)	0.1249 (4)	0.0482 (13)	
C43	0.7718 (6)	0.0410 (4)	0.0483 (4)	0.0487 (16)	
H43	0.7777	0.0954	−0.0040	0.058*	
C44	0.6507 (5)	−0.0938 (4)	−0.0010 (4)	0.0511 (16)	
H44A	0.6003	−0.0969	−0.0724	0.061*	
H44B	0.6884	−0.1568	0.0005	0.061*	
C45	0.5547 (5)	−0.1162 (5)	0.0651 (4)	0.0439 (15)	
S421	0.68139 (15)	0.21639 (13)	0.27511 (12)	0.0544 (5)	
C422	0.7658 (6)	0.1937 (5)	0.1848 (5)	0.0505 (16)	
N423	0.8776 (5)	0.2867 (4)	0.1773 (4)	0.0558 (14)	
C424	0.9084 (6)	0.3951 (6)	0.2437 (5)	0.0622 (18)	
O424	1.0021 (4)	0.4986 (4)	0.2506 (3)	0.0877 (15)	
C425	0.8101 (5)	0.3718 (5)	0.3113 (5)	0.0482 (15)	
C426	0.8307 (6)	0.4611 (5)	0.3868 (5)	0.0636 (18)	
H426	0.9061	0.5406	0.3996	0.076*	
C427	0.7337 (7)	0.4347 (6)	0.4514 (5)	0.0637 (19)	
O427	0.6309 (5)	0.3363 (4)	0.4379 (3)	0.0842 (15)	
O428	0.7767 (4)	0.5339 (4)	0.5256 (4)	0.0852 (14)	
C428	0.6931 (7)	0.5170 (6)	0.5969 (6)	0.094 (2)	
H48A	0.7096	0.4595	0.6416	0.112*	
H48B	0.5928	0.4809	0.5580	0.112*	
C429	0.7352 (7)	0.6415 (7)	0.6602 (6)	0.119 (3)	
H49A	0.6803	0.6314	0.7071	0.179*	
H49B	0.7184	0.6980	0.6155	0.179*	
H49C	0.8342	0.6763	0.6992	0.179*	
S451	0.3311 (4)	−0.2531 (3)	0.1357 (3)	0.0655 (10)	0.768 (6)
C452	0.4337 (6)	−0.2339 (5)	0.0544 (4)	0.0499 (15)	0.768 (6)
C453	0.398 (3)	−0.339 (2)	−0.0103 (19)	0.073 (3)	0.768 (6)
H453	0.4475	−0.3467	−0.0552	0.087*	0.768 (6)
C454	0.270 (2)	−0.4427 (17)	−0.002 (2)	0.075 (5)	0.768 (6)
H454	0.2228	−0.5220	−0.0449	0.090*	0.768 (6)
C455	0.2293 (14)	−0.4072 (9)	0.0772 (15)	0.073 (4)	0.768 (6)
H455	0.1540	−0.4614	0.0971	0.087*	0.768 (6)
S551	0.383 (3)	−0.3635 (19)	−0.0394 (18)	0.073 (3)	0.232 (6)
C552	0.4337 (6)	−0.2339 (5)	0.0544 (4)	0.0499 (15)	0.232 (6)
C553	0.349 (5)	−0.251 (4)	0.112 (4)	0.0655 (10)	0.232 (6)
H553	0.3652	−0.1924	0.1690	0.079*	0.232 (6)
C554	0.224 (5)	−0.375 (3)	0.073 (5)	0.073 (4)	0.232 (6)
H554	0.1462	−0.4003	0.0973	0.087*	0.232 (6)
C555	0.239 (9)	−0.446 (5)	−0.002 (8)	0.075 (5)	0.232 (6)
H555	0.1772	−0.5306	−0.0298	0.090*	0.232 (6)
O51	0.8569 (3)	−0.1355 (3)	0.1888 (3)	0.0513 (10)	
C51	0.8251 (6)	−0.1159 (6)	0.2805 (5)	0.0543 (16)	
C52	0.7268 (7)	−0.2241 (7)	0.3002 (6)	0.073 (2)	
C53	0.6939 (8)	−0.2041 (10)	0.3902 (8)	0.116 (3)	
H53	0.6310	−0.2738	0.4086	0.140*	
C54	0.7476 (11)	−0.0898 (13)	0.4529 (8)	0.132 (4)	
H54	0.7183	−0.0825	0.5107	0.158*	
C55	0.8442 (10)	0.0144 (10)	0.4315 (6)	0.105 (3)	
H55	0.8838	0.0924	0.4756	0.125*	
C56	0.8832 (6)	0.0023 (6)	0.3421 (5)	0.0704 (19)	
H56	0.9468	0.0725	0.3247	0.084*	
C57	0.6674 (7)	−0.3502 (6)	0.2333 (6)	0.105 (3)	
H57A	0.5900	−0.4086	0.2528	0.158*	
H57B	0.7399	−0.3803	0.2416	0.158*	
H57C	0.6337	−0.3435	0.1620	0.158*	

### Ethyl (Z)-2-{2-[3'-methyl-1'-phenyl-5-(thiophen-2-yl)-5'-(2-methylphenoxy)-3,4-dihydro-1'H,2H-3,4'-bipyrazole-2-yl]-4-oxo-4,5-dihydrothiazol-5-ylidene}acetate (II) Atomic displacement parameters (Å^2^)

**Table:**

	*U*^11^	*U*^22^	*U*^33^	*U*^12^	*U*^13^	*U*^23^
N1	0.042 (3)	0.044 (3)	0.045 (3)	0.016 (3)	0.016 (3)	0.008 (2)
N2	0.043 (3)	0.043 (3)	0.053 (3)	0.011 (2)	0.028 (3)	0.011 (3)
C3	0.047 (4)	0.041 (3)	0.046 (4)	0.017 (3)	0.025 (3)	0.007 (3)
C4	0.039 (4)	0.047 (3)	0.039 (4)	0.020 (3)	0.017 (3)	0.009 (3)
C5	0.040 (4)	0.042 (3)	0.036 (4)	0.007 (3)	0.016 (3)	0.000 (3)
C11	0.040 (4)	0.045 (3)	0.037 (4)	0.016 (3)	0.011 (3)	0.001 (3)
C12	0.050 (4)	0.048 (4)	0.065 (5)	0.014 (3)	0.016 (4)	0.009 (3)
C13	0.076 (5)	0.053 (4)	0.068 (5)	0.029 (4)	0.024 (4)	0.019 (4)
C14	0.062 (5)	0.075 (4)	0.058 (5)	0.032 (4)	0.010 (4)	0.015 (4)
C15	0.052 (4)	0.068 (4)	0.050 (5)	0.018 (4)	0.013 (4)	0.010 (4)
C16	0.051 (4)	0.050 (3)	0.035 (4)	0.015 (3)	0.020 (3)	0.015 (3)
C31	0.068 (4)	0.051 (3)	0.067 (5)	0.019 (3)	0.034 (4)	0.019 (3)
N41	0.047 (3)	0.042 (3)	0.047 (3)	0.015 (2)	0.021 (3)	0.012 (2)
N42	0.047 (3)	0.046 (3)	0.057 (4)	0.022 (2)	0.022 (3)	0.006 (3)
C43	0.057 (4)	0.051 (3)	0.043 (4)	0.021 (3)	0.027 (3)	0.003 (3)
C44	0.043 (4)	0.061 (4)	0.046 (4)	0.021 (3)	0.014 (3)	0.001 (3)
C45	0.047 (4)	0.047 (3)	0.041 (4)	0.024 (3)	0.013 (3)	0.013 (3)
S421	0.0588 (11)	0.0465 (8)	0.0573 (12)	0.0175 (8)	0.0263 (9)	0.0045 (8)
C422	0.063 (4)	0.051 (4)	0.052 (4)	0.034 (3)	0.027 (4)	0.013 (3)
N423	0.059 (3)	0.042 (3)	0.061 (4)	0.009 (2)	0.030 (3)	0.005 (3)
C424	0.061 (5)	0.055 (4)	0.058 (5)	0.014 (4)	0.018 (4)	0.007 (4)
O424	0.087 (3)	0.056 (2)	0.089 (4)	−0.008 (2)	0.043 (3)	−0.005 (3)
C425	0.056 (4)	0.038 (3)	0.051 (4)	0.017 (3)	0.021 (3)	0.010 (3)
C426	0.074 (5)	0.053 (4)	0.064 (5)	0.020 (3)	0.035 (4)	0.001 (4)
C427	0.074 (5)	0.053 (4)	0.052 (5)	0.030 (4)	0.001 (4)	−0.013 (4)
O427	0.093 (4)	0.071 (3)	0.074 (4)	0.018 (3)	0.033 (3)	−0.005 (3)
O428	0.101 (4)	0.074 (3)	0.075 (4)	0.030 (3)	0.036 (3)	−0.011 (3)
C428	0.102 (6)	0.107 (6)	0.076 (6)	0.048 (5)	0.033 (5)	−0.006 (5)
C429	0.147 (7)	0.131 (6)	0.083 (6)	0.073 (6)	0.028 (5)	−0.020 (5)
S451	0.0732 (19)	0.0564 (14)	0.068 (2)	0.0167 (12)	0.0389 (13)	0.0163 (14)
C452	0.058 (4)	0.040 (3)	0.048 (4)	0.017 (3)	0.018 (3)	0.004 (3)
C453	0.085 (6)	0.043 (7)	0.053 (11)	0.002 (5)	0.007 (7)	−0.003 (5)
C454	0.080 (13)	0.046 (4)	0.066 (6)	−0.001 (5)	0.017 (9)	−0.002 (4)
C455	0.083 (5)	0.046 (7)	0.079 (6)	0.016 (5)	0.026 (5)	0.014 (7)
S551	0.085 (6)	0.043 (7)	0.053 (11)	0.002 (5)	0.007 (7)	−0.003 (5)
C552	0.058 (4)	0.040 (3)	0.048 (4)	0.017 (3)	0.018 (3)	0.004 (3)
C553	0.0732 (19)	0.0564 (14)	0.068 (2)	0.0167 (12)	0.0389 (13)	0.0163 (14)
C554	0.083 (5)	0.046 (7)	0.079 (6)	0.016 (5)	0.026 (5)	0.014 (7)
C555	0.080 (13)	0.046 (4)	0.066 (6)	−0.001 (5)	0.017 (9)	−0.002 (4)
O51	0.049 (2)	0.056 (2)	0.048 (3)	0.0145 (19)	0.024 (2)	0.010 (2)
C51	0.058 (4)	0.075 (4)	0.042 (5)	0.037 (4)	0.020 (4)	0.020 (4)
C52	0.065 (5)	0.110 (6)	0.068 (6)	0.041 (5)	0.042 (4)	0.065 (5)
C53	0.098 (7)	0.169 (9)	0.110 (10)	0.054 (7)	0.068 (7)	0.081 (7)
C54	0.140 (10)	0.248 (15)	0.078 (8)	0.125 (10)	0.068 (7)	0.074 (9)
C55	0.136 (8)	0.182 (9)	0.049 (6)	0.107 (7)	0.047 (5)	0.035 (6)
C56	0.077 (5)	0.102 (5)	0.055 (5)	0.052 (4)	0.034 (4)	0.019 (4)
C57	0.092 (6)	0.082 (5)	0.129 (7)	0.013 (4)	0.041 (5)	0.070 (5)

### Ethyl (Z)-2-{2-[3'-methyl-1'-phenyl-5-(thiophen-2-yl)-5'-(2-methylphenoxy)-3,4-dihydro-1'H,2H-3,4'-bipyrazole-2-yl]-4-oxo-4,5-dihydrothiazol-5-ylidene}acetate (II) Geometric parameters (Å, º)

**Table:**

N1—C5	1.358 (6)	C426—C427	1.489 (8)
N1—N2	1.374 (5)	C426—H426	0.9300
N1—C11	1.426 (6)	C427—O427	1.203 (6)
N2—C3	1.333 (6)	C427—O428	1.327 (6)
C3—C4	1.409 (6)	O428—C428	1.458 (7)
C3—C31	1.497 (6)	C428—C429	1.477 (7)
C4—C5	1.364 (7)	C428—H48A	0.9700
C4—C43	1.485 (6)	C428—H48B	0.9700
C5—O51	1.367 (6)	C429—H49A	0.9600
C11—C16	1.382 (6)	C429—H49B	0.9600
C11—C12	1.382 (7)	C429—H49C	0.9600
C12—C13	1.390 (7)	S451—C455	1.699 (9)
C12—H12	0.9300	S451—C452	1.723 (6)
C13—C14	1.384 (7)	C452—C453	1.320 (16)
C13—H13	0.9300	C453—C454	1.47 (2)
C14—C15	1.367 (7)	C453—H453	0.9300
C14—H14	0.9300	C454—C455	1.359 (10)
C15—C16	1.377 (7)	C454—H454	0.9300
C15—H15	0.9300	C455—H455	0.9300
C16—H16	0.9300	S551—C555	1.698 (14)
C31—H31A	0.9600	C553—C554	1.48 (2)
C31—H31B	0.9600	C553—H553	0.9300
C31—H31C	0.9600	C554—C555	1.359 (13)
N41—C45	1.293 (5)	C554—H554	0.9300
N41—N42	1.405 (5)	C555—H555	0.9300
N42—C422	1.333 (5)	O51—C51	1.405 (6)
N42—C43	1.507 (6)	C51—C56	1.379 (7)
C43—C44	1.557 (6)	C51—C52	1.387 (8)
C43—H43	0.9800	C52—C53	1.390 (10)
C44—C45	1.499 (7)	C52—C57	1.481 (8)
C44—H44A	0.9700	C53—C54	1.355 (10)
C44—H44B	0.9700	C53—H53	0.9300
C45—C452	1.441 (6)	C54—C55	1.362 (11)
S421—C425	1.736 (5)	C54—H54	0.9300
S421—C422	1.771 (5)	C55—C56	1.400 (9)
C422—N423	1.300 (6)	C55—H55	0.9300
N423—C424	1.378 (6)	C56—H56	0.9300
C424—O424	1.211 (6)	C57—H57A	0.9600
C424—C425	1.534 (7)	C57—H57B	0.9600
C425—C426	1.325 (6)	C57—H57C	0.9600
			
C5—N1—N2	109.6 (4)	C424—C425—S421	109.7 (4)
C5—N1—C11	131.7 (5)	C425—C426—C427	120.3 (5)
N2—N1—C11	118.6 (4)	C425—C426—H426	119.9
C3—N2—N1	105.2 (4)	C427—C426—H426	119.9
N2—C3—C4	112.1 (5)	O427—C427—O428	125.0 (7)
N2—C3—C31	120.0 (5)	O427—C427—C426	124.2 (6)
C4—C3—C31	127.9 (6)	O428—C427—C426	110.7 (6)
C5—C4—C3	103.6 (5)	C427—O428—C428	115.7 (5)
C5—C4—C43	128.2 (5)	O428—C428—C429	108.9 (6)
C3—C4—C43	128.2 (5)	O428—C428—H48A	109.9
N1—C5—C4	109.4 (5)	C429—C428—H48A	109.9
N1—C5—O51	122.3 (5)	O428—C428—H48B	109.9
C4—C5—O51	128.2 (5)	C429—C428—H48B	109.9
C16—C11—C12	119.6 (5)	H48A—C428—H48B	108.3
C16—C11—N1	119.3 (5)	C428—C429—H49A	109.5
C12—C11—N1	121.1 (5)	C428—C429—H49B	109.5
C11—C12—C13	119.0 (5)	H49A—C429—H49B	109.5
C11—C12—H12	120.5	C428—C429—H49C	109.5
C13—C12—H12	120.5	H49A—C429—H49C	109.5
C14—C13—C12	121.4 (6)	H49B—C429—H49C	109.5
C14—C13—H13	119.3	C455—S451—C452	91.1 (5)
C12—C13—H13	119.3	C453—C452—C45	124.6 (12)
C15—C14—C13	118.5 (6)	C453—C452—S451	113.6 (11)
C15—C14—H14	120.7	C45—C452—S451	121.6 (4)
C13—C14—H14	120.7	C452—C453—C454	111.2 (14)
C14—C15—C16	121.0 (6)	C452—C453—H453	124.4
C14—C15—H15	119.5	C454—C453—H453	124.4
C16—C15—H15	119.5	C455—C454—C453	111.1 (10)
C15—C16—C11	120.4 (5)	C455—C454—H454	124.5
C15—C16—H16	119.8	C453—C454—H454	124.5
C11—C16—H16	119.8	C454—C455—S451	112.8 (10)
C3—C31—H31A	109.5	C454—C455—H455	123.6
C3—C31—H31B	109.5	S451—C455—H455	123.6
H31A—C31—H31B	109.5	C554—C553—H553	124.6
C3—C31—H31C	109.5	C555—C554—C553	111.3 (16)
H31A—C31—H31C	109.5	C555—C554—H554	124.4
H31B—C31—H31C	109.5	C553—C554—H554	124.4
C45—N41—N42	107.5 (4)	C554—C555—S551	112.2 (17)
C422—N42—N41	120.5 (4)	C554—C555—H555	123.9
C422—N42—C43	125.3 (4)	S551—C555—H555	123.9
N41—N42—C43	114.2 (4)	C5—O51—C51	117.5 (4)
C4—C43—N42	112.5 (4)	C56—C51—C52	123.9 (6)
C4—C43—C44	115.7 (4)	C56—C51—O51	122.0 (6)
N42—C43—C44	98.8 (4)	C52—C51—O51	114.0 (6)
C4—C43—H43	109.8	C51—C52—C53	114.0 (7)
N42—C43—H43	109.8	C51—C52—C57	122.7 (7)
C44—C43—H43	109.8	C53—C52—C57	123.3 (7)
C45—C44—C43	104.6 (4)	C54—C53—C52	124.3 (10)
C45—C44—H44A	110.8	C54—C53—H53	117.8
C43—C44—H44A	110.8	C52—C53—H53	117.8
C45—C44—H44B	110.8	C53—C54—C55	120.1 (11)
C43—C44—H44B	110.8	C53—C54—H54	119.9
H44A—C44—H44B	108.9	C55—C54—H54	119.9
N41—C45—C452	121.4 (5)	C54—C55—C56	119.1 (9)
N41—C45—C44	114.0 (5)	C54—C55—H55	120.5
C452—C45—C44	124.5 (5)	C56—C55—H55	120.5
C425—S421—C422	87.3 (3)	C51—C56—C55	118.5 (7)
N423—C422—N42	121.8 (5)	C51—C56—H56	120.7
N423—C422—S421	120.3 (4)	C55—C56—H56	120.7
N42—C422—S421	117.9 (4)	C52—C57—H57A	109.5
C422—N423—C424	110.0 (5)	C52—C57—H57B	109.5
O424—C424—N423	125.4 (6)	H57A—C57—H57B	109.5
O424—C424—C425	122.0 (6)	C52—C57—H57C	109.5
N423—C424—C425	112.5 (5)	H57A—C57—H57C	109.5
C426—C425—C424	121.8 (5)	H57B—C57—H57C	109.5
C426—C425—S421	128.4 (5)		
			
C5—N1—N2—C3	−0.1 (5)	C43—N42—C422—S421	−176.4 (4)
C11—N1—N2—C3	−178.6 (4)	C425—S421—C422—N423	−0.2 (5)
N1—N2—C3—C4	−1.0 (5)	C425—S421—C422—N42	−179.4 (4)
N1—N2—C3—C31	178.2 (4)	N42—C422—N423—C424	176.8 (5)
N2—C3—C4—C5	1.6 (5)	S421—C422—N423—C424	−2.4 (7)
C31—C3—C4—C5	−177.5 (5)	C422—N423—C424—O424	−176.2 (6)
N2—C3—C4—C43	−178.3 (4)	C422—N423—C424—C425	4.1 (7)
C31—C3—C4—C43	2.7 (8)	O424—C424—C425—C426	−6.1 (9)
N2—N1—C5—C4	1.1 (5)	N423—C424—C425—C426	173.5 (5)
C11—N1—C5—C4	179.4 (4)	O424—C424—C425—S421	176.0 (5)
N2—N1—C5—O51	177.6 (4)	N423—C424—C425—S421	−4.3 (6)
C11—N1—C5—O51	−4.2 (7)	C422—S421—C425—C426	−175.3 (6)
C3—C4—C5—N1	−1.6 (5)	C422—S421—C425—C424	2.4 (4)
C43—C4—C5—N1	178.3 (4)	C424—C425—C426—C427	−179.5 (6)
C3—C4—C5—O51	−177.8 (5)	S421—C425—C426—C427	−2.0 (8)
C43—C4—C5—O51	2.1 (8)	C425—C426—C427—O427	−2.8 (10)
C5—N1—C11—C16	−160.7 (5)	C425—C426—C427—O428	176.9 (5)
N2—N1—C11—C16	17.5 (6)	O427—C427—O428—C428	2.3 (9)
C5—N1—C11—C12	18.0 (7)	C426—C427—O428—C428	−177.4 (5)
N2—N1—C11—C12	−163.8 (4)	C427—O428—C428—C429	−168.6 (5)
C16—C11—C12—C13	0.2 (8)	N41—C45—C452—C453	−177.0 (19)
N1—C11—C12—C13	−178.5 (4)	C44—C45—C452—C453	5 (2)
C11—C12—C13—C14	−0.4 (8)	N41—C45—C452—S451	−3.3 (7)
C12—C13—C14—C15	−0.3 (9)	C44—C45—C452—S451	178.4 (5)
C13—C14—C15—C16	1.3 (9)	C455—S451—C452—C453	−2.9 (18)
C14—C15—C16—C11	−1.6 (8)	C455—S451—C452—C45	−177.2 (8)
C12—C11—C16—C15	0.8 (7)	C45—C452—C453—C454	180 (2)
N1—C11—C16—C15	179.5 (5)	S451—C452—C453—C454	5 (3)
C45—N41—N42—C422	178.4 (5)	C452—C453—C454—C455	−6 (4)
C45—N41—N42—C43	−4.1 (6)	C453—C454—C455—S451	4 (4)
C5—C4—C43—N42	70.7 (6)	C452—S451—C455—C454	−1 (2)
C3—C4—C43—N42	−109.5 (6)	C553—C554—C555—S551	−8 (12)
C5—C4—C43—C44	−41.9 (7)	N1—C5—O51—C51	92.8 (5)
C3—C4—C43—C44	138.0 (5)	C4—C5—O51—C51	−91.4 (6)
C422—N42—C43—C4	62.7 (6)	C5—O51—C51—C56	−0.8 (7)
N41—N42—C43—C4	−114.6 (5)	C5—O51—C51—C52	176.7 (5)
C422—N42—C43—C44	−174.7 (5)	C56—C51—C52—C53	−1.7 (9)
N41—N42—C43—C44	8.0 (5)	O51—C51—C52—C53	−179.1 (5)
C4—C43—C44—C45	111.9 (5)	C56—C51—C52—C57	−179.3 (6)
N42—C43—C44—C45	−8.3 (5)	O51—C51—C52—C57	3.3 (8)
N42—N41—C45—C452	179.2 (5)	C51—C52—C53—C54	2.1 (12)
N42—N41—C45—C44	−2.3 (6)	C57—C52—C53—C54	179.6 (8)
C43—C44—C45—N41	7.3 (6)	C52—C53—C54—C55	−2.4 (15)
C43—C44—C45—C452	−174.3 (5)	C53—C54—C55—C56	2.2 (14)
N41—N42—C422—N423	−178.4 (5)	C52—C51—C56—C55	1.7 (9)
C43—N42—C422—N423	4.4 (8)	O51—C51—C56—C55	178.9 (5)
N41—N42—C422—S421	0.7 (6)	C54—C55—C56—C51	−1.9 (11)

### Ethyl (Z)-2-{2-[3'-methyl-1'-phenyl-5-(thiophen-2-yl)-5'-(2-methylphenoxy)-3,4-dihydro-1'H,2H-3,4'-bipyrazole-2-yl]-4-oxo-4,5-dihydrothiazol-5-ylidene}acetate (II) Hydrogen-bond geometry (Å, º)

**Table:**

*D*—H···*A*	*D*—H	H···*A*	*D*···*A*	*D*—H···*A*
C13—H13···O424^i^	0.93	2.49	3.200 (7)	133
C54—H54···*Cg*1^ii^	0.93	2.91	3.714 (12)	146
C553—H553···*Cg*1^iii^	0.93	2.92	3.76 (5)	151

Symmetry codes: (i) *x*, *y*−1, *z*; (ii) −*x*+2, −*y*, −*z*+1; (iii) *x*−1, *y*, *z*.

### 4-(4-Bromophenyl)-2-[5'-(2,4-dichlorophenoxy)-3'-methyl-1'-phenyl-5-(thiophen-2-yl)-3,4-dihydro-1'H,2H-3,4'-bipyrazole-2-yl]-4-thiazole (III) Crystal data

**Table:**

C_32_H_22_BrCl_2_N_5_OS_2_	*Z* = 2
*M**_r_* = 707.47	*F*(000) = 716
Triclinic, *P*1	*D*_x_ = 1.517 Mg m^−^^3^
*a* = 12.3200 (9) Å	Mo *K*α radiation, λ = 0.71073 Å
*b* = 12.5700 (9) Å	Cell parameters from 6664 reflections
*c* = 12.7742 (9) Å	θ = 2.6–27.8°
α = 117.202 (8)°	µ = 1.67 mm^−^^1^
β = 102.879 (7)°	*T* = 296 K
γ = 105.727 (7)°	Plate, yellow
*V* = 1548.4 (2) Å^3^	0.40 × 0.40 × 0.08 mm

### 4-(4-Bromophenyl)-2-[5'-(2,4-dichlorophenoxy)-3'-methyl-1'-phenyl-5-(thiophen-2-yl)-3,4-dihydro-1'H,2H-3,4'-bipyrazole-2-yl]-4-thiazole (III) Data collection

**Table:**

Oxford Diffraction Xcalibur with Sapphire CCD detector diffractometer	5773 independent reflections
Radiation source: Enhance (Mo) X-ray Source	3158 reflections with *I* > 2σ(*I*)
Graphite monochromator	*R*_int_ = 0.022
ω scans	θ_max_ = 25.6°, θ_min_ = 2.6°
Absorption correction: multi-scan (CrysAlis RED; Oxford Diffraction, 2009)	*h* = −13→14
*T*_min_ = 0.779, *T*_max_ = 0.875	*k* = −15→10
10549 measured reflections	*l* = −10→15

### 4-(4-Bromophenyl)-2-[5'-(2,4-dichlorophenoxy)-3'-methyl-1'-phenyl-5-(thiophen-2-yl)-3,4-dihydro-1'H,2H-3,4'-bipyrazole-2-yl]-4-thiazole (III) Refinement

**Table:**

Refinement on *F*^2^	Primary atom site location: difference Fourier map
Least-squares matrix: full	Hydrogen site location: inferred from neighbouring sites
*R*[*F*^2^ > 2σ(*F*^2^)] = 0.039	H-atom parameters constrained
*wR*(*F*^2^) = 0.095	*w* = 1/[σ^2^(*F*_o_^2^) + (0.0475*P*)^2^] where *P* = (*F*_o_^2^ + 2*F*_c_^2^)/3
*S* = 0.93	(Δ/σ)_max_ < 0.001
5773 reflections	Δρ_max_ = 0.47 e Å^−^^3^
402 parameters	Δρ_min_ = −0.34 e Å^−^^3^
10 restraints	

### 4-(4-Bromophenyl)-2-[5'-(2,4-dichlorophenoxy)-3'-methyl-1'-phenyl-5-(thiophen-2-yl)-3,4-dihydro-1'H,2H-3,4'-bipyrazole-2-yl]-4-thiazole (III) Special details

**Table:**

Experimental. CrysAlis RED, Oxford Diffraction Ltd., 2009 Empirical absorption correction using spherical harmonics, implemented in SCALE3 ABSPACK scaling algorithm.
Geometry. All esds (except the esd in the dihedral angle between two l.s. planes) are estimated using the full covariance matrix. The cell esds are taken into account individually in the estimation of esds in distances, angles and torsion angles; correlations between esds in cell parameters are only used when they are defined by crystal symmetry. An approximate (isotropic) treatment of cell esds is used for estimating esds involving l.s. planes.

### 4-(4-Bromophenyl)-2-[5'-(2,4-dichlorophenoxy)-3'-methyl-1'-phenyl-5-(thiophen-2-yl)-3,4-dihydro-1'H,2H-3,4'-bipyrazole-2-yl]-4-thiazole (III) Fractional atomic coordinates and isotropic or equivalent isotropic displacement parameters (Å^2^)

**Table:**

	*x*	*y*	*z*	*U*_iso_*/*U*_eq_	Occ. (<1)
N1	0.5457 (2)	0.3735 (2)	0.86216 (19)	0.0478 (5)	
N2	0.4327 (2)	0.3754 (2)	0.8277 (2)	0.0514 (6)	
C3	0.4368 (3)	0.4319 (2)	0.7616 (2)	0.0495 (7)	
C4	0.5508 (3)	0.4674 (2)	0.7522 (2)	0.0477 (7)	
C5	0.6162 (2)	0.4278 (2)	0.8157 (2)	0.0446 (6)	
C11	0.5693 (3)	0.3119 (2)	0.9294 (2)	0.0449 (6)	
C12	0.6877 (3)	0.3383 (3)	0.9963 (3)	0.0552 (7)	
H12	0.7546	0.3982	0.9997	0.066*	
C13	0.7064 (3)	0.2750 (3)	1.0586 (3)	0.0591 (8)	
H13	0.7863	0.2915	1.1029	0.071*	
C14	0.6089 (3)	0.1887 (3)	1.0558 (3)	0.0630 (8)	
H14	0.6221	0.1465	1.0978	0.076*	
C15	0.4917 (3)	0.1647 (3)	0.9907 (3)	0.0719 (9)	
H15	0.4252	0.1064	0.9893	0.086*	
C16	0.4707 (3)	0.2257 (3)	0.9271 (3)	0.0617 (8)	
H16	0.3906	0.2088	0.8830	0.074*	
C31	0.3270 (3)	0.4476 (3)	0.7047 (3)	0.0686 (8)	
H31A	0.3053	0.4075	0.6140	0.103*	
H31B	0.3464	0.5402	0.7453	0.103*	
H31C	0.2589	0.4054	0.7181	0.103*	
N41	0.7375 (3)	0.5162 (3)	0.5905 (3)	0.0661 (7)	
N42	0.6211 (2)	0.4547 (3)	0.5794 (2)	0.0670 (7)	
C43	0.5928 (3)	0.5358 (3)	0.6881 (3)	0.0576 (7)	
H43	0.5288	0.5591	0.6567	0.069*	
C44	0.7168 (3)	0.6608 (3)	0.7729 (3)	0.0672 (8)	
H44A	0.7058	0.7390	0.7866	0.081*	
H44B	0.7546	0.6741	0.8558	0.081*	
C45	0.7931 (3)	0.6315 (3)	0.6965 (3)	0.0622 (8)	
S421	0.59658 (8)	0.25102 (9)	0.35914 (8)	0.0776 (3)	
C422	0.5442 (3)	0.3311 (3)	0.4739 (3)	0.0576 (8)	
N423	0.4313 (2)	0.2671 (2)	0.4522 (2)	0.0554 (6)	
C424	0.3781 (3)	0.1417 (3)	0.3379 (3)	0.0568 (7)	
C425	0.4545 (3)	0.1182 (3)	0.2773 (3)	0.0724 (9)	
H425	0.4325	0.0394	0.2004	0.087*	
C441	0.2509 (3)	0.0523 (3)	0.2964 (3)	0.0557 (7)	
C442	0.1828 (3)	0.0877 (3)	0.3669 (3)	0.0663 (8)	
H442	0.2188	0.1712	0.4429	0.080*	
C443	0.0635 (3)	0.0037 (3)	0.3287 (3)	0.0738 (9)	
H443	0.0196	0.0310	0.3783	0.089*	
C444	0.0085 (3)	−0.1208 (3)	0.2172 (3)	0.0667 (8)	
Br44	−0.15403 (3)	−0.23859 (4)	0.16704 (4)	0.09399 (17)	
C445	0.0740 (4)	−0.1594 (3)	0.1453 (3)	0.0803 (10)	
H445	0.0378	−0.2435	0.0700	0.096*	
C446	0.1919 (4)	−0.0751 (3)	0.1836 (3)	0.0778 (9)	
H446	0.2349	−0.1030	0.1331	0.093*	
S451	0.99714 (13)	0.68706 (13)	0.64070 (13)	0.1055 (6)	0.947 (4)
C452	0.9177 (3)	0.7199 (4)	0.7353 (4)	0.0739 (9)	0.947 (4)
C453	0.9844 (6)	0.8384 (6)	0.8487 (6)	0.0934 (17)	0.947 (4)
H453	0.9561	0.8733	0.9127	0.112*	0.947 (4)
C454	1.1069 (5)	0.9043 (5)	0.8577 (6)	0.1124 (18)	0.947 (4)
H454	1.1665	0.9865	0.9289	0.135*	0.947 (4)
C455	1.1232 (5)	0.8339 (6)	0.7532 (7)	0.1145 (18)	0.947 (4)
H455	1.1955	0.8612	0.7421	0.137*	0.947 (4)
S551	1.025 (3)	0.871 (2)	0.863 (3)	0.0934 (17)	0.053 (4)
C552	0.9177 (3)	0.7199 (4)	0.7353 (4)	0.0739 (9)	0.053 (4)
C553	0.966 (5)	0.658 (5)	0.654 (6)	0.1055 (6)	0.053 (4)
H553	0.9264	0.5696	0.5857	0.127*	0.053 (4)
C554	1.090 (5)	0.750 (6)	0.687 (7)	0.1145 (18)	0.053 (4)
H554	1.1374	0.7270	0.6403	0.137*	0.053 (4)
C555	1.126 (7)	0.868 (8)	0.790 (10)	0.1124 (18)	0.053 (4)
H555	1.1972	0.9419	0.8192	0.135*	0.053 (4)
O51	0.73172 (16)	0.43438 (16)	0.83109 (15)	0.0493 (4)	
C51	0.7387 (2)	0.3304 (2)	0.7303 (2)	0.0440 (6)	
C52	0.8521 (2)	0.3502 (3)	0.7240 (3)	0.0488 (7)	
Cl52	0.97805 (7)	0.50033 (8)	0.83960 (9)	0.0895 (3)	
C53	0.8662 (3)	0.2526 (3)	0.6269 (3)	0.0586 (8)	
H53	0.9429	0.2664	0.6230	0.070*	
C54	0.7648 (3)	0.1344 (3)	0.5356 (3)	0.0563 (7)	
Cl54	0.77954 (9)	0.01019 (9)	0.41070 (8)	0.0873 (3)	
C55	0.6533 (3)	0.1136 (3)	0.5414 (3)	0.0634 (8)	
H55	0.5856	0.0330	0.4793	0.076*	
C56	0.6397 (3)	0.2115 (3)	0.6391 (3)	0.0576 (7)	
H56	0.5628	0.1966	0.6428	0.069*	

### 4-(4-Bromophenyl)-2-[5'-(2,4-dichlorophenoxy)-3'-methyl-1'-phenyl-5-(thiophen-2-yl)-3,4-dihydro-1'H,2H-3,4'-bipyrazole-2-yl]-4-thiazole (III) Atomic displacement parameters (Å^2^)

**Table:**

	*U*^11^	*U*^22^	*U*^33^	*U*^12^	*U*^13^	*U*^23^
N1	0.0546 (15)	0.0491 (13)	0.0455 (13)	0.0245 (12)	0.0228 (11)	0.0279 (12)
N2	0.0558 (15)	0.0592 (14)	0.0494 (14)	0.0323 (12)	0.0271 (12)	0.0304 (12)
C3	0.0627 (19)	0.0521 (17)	0.0406 (16)	0.0336 (15)	0.0240 (14)	0.0241 (14)
C4	0.0656 (19)	0.0472 (16)	0.0404 (16)	0.0306 (15)	0.0266 (14)	0.0257 (14)
C5	0.0547 (18)	0.0403 (15)	0.0406 (15)	0.0226 (14)	0.0252 (14)	0.0198 (13)
C11	0.0557 (18)	0.0434 (15)	0.0406 (15)	0.0250 (14)	0.0224 (14)	0.0235 (13)
C12	0.062 (2)	0.0595 (18)	0.0545 (17)	0.0272 (16)	0.0278 (15)	0.0363 (16)
C13	0.065 (2)	0.070 (2)	0.0525 (18)	0.0350 (18)	0.0262 (16)	0.0367 (17)
C14	0.090 (2)	0.060 (2)	0.0539 (19)	0.0401 (19)	0.0315 (18)	0.0366 (16)
C15	0.079 (2)	0.068 (2)	0.080 (2)	0.0250 (19)	0.0351 (19)	0.0506 (19)
C16	0.0580 (18)	0.066 (2)	0.067 (2)	0.0224 (17)	0.0225 (16)	0.0438 (18)
C31	0.085 (2)	0.083 (2)	0.065 (2)	0.054 (2)	0.0393 (18)	0.0460 (18)
N41	0.080 (2)	0.0741 (19)	0.0642 (18)	0.0358 (17)	0.0382 (16)	0.0471 (17)
N42	0.0780 (19)	0.0714 (18)	0.0550 (17)	0.0272 (16)	0.0397 (15)	0.0343 (15)
C43	0.074 (2)	0.0604 (19)	0.0558 (18)	0.0365 (18)	0.0330 (17)	0.0377 (16)
C44	0.089 (2)	0.0558 (19)	0.066 (2)	0.0305 (19)	0.0310 (19)	0.0409 (17)
C45	0.078 (2)	0.071 (2)	0.068 (2)	0.039 (2)	0.036 (2)	0.054 (2)
S421	0.0958 (7)	0.0867 (6)	0.0679 (5)	0.0459 (6)	0.0536 (5)	0.0425 (5)
C422	0.080 (2)	0.067 (2)	0.0479 (19)	0.0399 (19)	0.0355 (17)	0.0392 (18)
N423	0.0729 (18)	0.0614 (16)	0.0449 (14)	0.0360 (15)	0.0317 (13)	0.0311 (13)
C424	0.080 (2)	0.062 (2)	0.0451 (18)	0.0416 (19)	0.0316 (17)	0.0329 (17)
C425	0.095 (2)	0.072 (2)	0.059 (2)	0.044 (2)	0.0440 (19)	0.0326 (17)
C441	0.081 (2)	0.0597 (19)	0.0423 (17)	0.0442 (18)	0.0298 (16)	0.0295 (15)
C442	0.078 (2)	0.060 (2)	0.0572 (19)	0.041 (2)	0.0311 (18)	0.0224 (16)
C443	0.077 (2)	0.070 (2)	0.070 (2)	0.042 (2)	0.0344 (19)	0.0274 (19)
C444	0.076 (2)	0.069 (2)	0.068 (2)	0.0457 (19)	0.0303 (19)	0.0385 (19)
Br44	0.0795 (3)	0.0793 (3)	0.1006 (3)	0.0331 (2)	0.0273 (2)	0.0381 (2)
C445	0.092 (3)	0.061 (2)	0.064 (2)	0.031 (2)	0.031 (2)	0.0186 (18)
C446	0.101 (3)	0.078 (3)	0.061 (2)	0.048 (2)	0.048 (2)	0.031 (2)
S451	0.0958 (12)	0.1464 (12)	0.1214 (10)	0.0581 (10)	0.0620 (8)	0.0961 (9)
C452	0.074 (2)	0.078 (2)	0.088 (3)	0.030 (2)	0.028 (2)	0.062 (2)
C453	0.067 (4)	0.080 (4)	0.124 (4)	0.012 (3)	0.028 (3)	0.065 (3)
C454	0.076 (4)	0.112 (4)	0.155 (5)	0.026 (3)	0.034 (3)	0.091 (4)
C455	0.075 (3)	0.149 (5)	0.171 (6)	0.044 (4)	0.046 (4)	0.128 (5)
S551	0.067 (4)	0.080 (4)	0.124 (4)	0.012 (3)	0.028 (3)	0.065 (3)
C552	0.074 (2)	0.078 (2)	0.088 (3)	0.030 (2)	0.028 (2)	0.062 (2)
C553	0.0958 (12)	0.1464 (12)	0.1214 (10)	0.0581 (10)	0.0620 (8)	0.0961 (9)
C554	0.075 (3)	0.149 (5)	0.171 (6)	0.044 (4)	0.046 (4)	0.128 (5)
C555	0.076 (4)	0.112 (4)	0.155 (5)	0.026 (3)	0.034 (3)	0.091 (4)
O51	0.0512 (12)	0.0460 (11)	0.0451 (11)	0.0196 (9)	0.0208 (9)	0.0212 (9)
C51	0.0533 (18)	0.0436 (16)	0.0432 (16)	0.0229 (15)	0.0241 (14)	0.0266 (14)
C52	0.0421 (16)	0.0517 (17)	0.0502 (17)	0.0159 (14)	0.0166 (14)	0.0299 (15)
Cl52	0.0503 (5)	0.0766 (6)	0.0886 (6)	0.0101 (4)	0.0132 (4)	0.0238 (5)
C53	0.0549 (19)	0.077 (2)	0.064 (2)	0.0381 (18)	0.0339 (17)	0.0431 (19)
C54	0.073 (2)	0.062 (2)	0.0535 (18)	0.0405 (18)	0.0372 (17)	0.0341 (17)
Cl54	0.1139 (7)	0.0907 (6)	0.0691 (5)	0.0620 (6)	0.0543 (5)	0.0350 (5)
C55	0.063 (2)	0.0437 (17)	0.064 (2)	0.0156 (15)	0.0306 (16)	0.0180 (15)
C56	0.0493 (17)	0.0473 (18)	0.068 (2)	0.0167 (15)	0.0322 (16)	0.0242 (16)

### 4-(4-Bromophenyl)-2-[5'-(2,4-dichlorophenoxy)-3'-methyl-1'-phenyl-5-(thiophen-2-yl)-3,4-dihydro-1'H,2H-3,4'-bipyrazole-2-yl]-4-thiazole (III) Geometric parameters (Å, º)

**Table:**

N1—C5	1.361 (3)	C425—H425	0.9300
N1—N2	1.374 (3)	C441—C442	1.373 (4)
N1—C11	1.428 (3)	C441—C446	1.400 (4)
N2—C3	1.330 (3)	C442—C443	1.372 (4)
C3—C4	1.404 (3)	C442—H442	0.9300
C3—C31	1.495 (3)	C443—C444	1.377 (4)
C4—C5	1.361 (3)	C443—H443	0.9300
C4—C43	1.506 (3)	C444—C445	1.366 (4)
C5—O51	1.367 (3)	C444—Br44	1.888 (3)
C11—C12	1.375 (3)	C445—C446	1.359 (4)
C11—C16	1.375 (3)	C445—H445	0.9300
C12—C13	1.385 (3)	C446—H446	0.9300
C12—H12	0.9300	S451—C452	1.696 (4)
C13—C14	1.364 (4)	S451—C455	1.706 (6)
C13—H13	0.9300	C452—C453	1.351 (7)
C14—C15	1.366 (4)	C453—C454	1.461 (10)
C14—H14	0.9300	C453—H453	0.9300
C15—C16	1.379 (4)	C454—C455	1.321 (7)
C15—H15	0.9300	C454—H454	0.9300
C16—H16	0.9300	C455—H455	0.9300
C31—H31A	0.9600	S551—C555	1.707 (12)
C31—H31B	0.9600	C553—C554	1.461 (14)
C31—H31C	0.9600	C553—H553	0.9300
N41—C45	1.287 (4)	C554—C555	1.322 (12)
N41—N42	1.372 (3)	C554—H554	0.9300
N42—C422	1.359 (4)	C555—H555	0.9300
N42—C43	1.484 (3)	O51—C51	1.395 (3)
C43—C44	1.543 (4)	C51—C56	1.366 (3)
C43—H43	0.9800	C51—C52	1.379 (3)
C44—C45	1.496 (4)	C52—C53	1.377 (4)
C44—H44A	0.9700	C52—Cl52	1.730 (3)
C44—H44B	0.9700	C53—C54	1.374 (4)
C45—C452	1.439 (4)	C53—H53	0.9300
S421—C425	1.717 (3)	C54—C55	1.353 (4)
S421—C422	1.734 (3)	C54—Cl54	1.737 (3)
C422—N423	1.295 (3)	C55—C56	1.379 (4)
N423—C424	1.399 (3)	C55—H55	0.9300
C424—C425	1.356 (4)	C56—H56	0.9300
C424—C441	1.460 (4)		
			
C5—N1—N2	109.84 (19)	C425—C424—C441	127.3 (3)
C5—N1—C11	130.5 (2)	N423—C424—C441	118.8 (3)
N2—N1—C11	119.5 (2)	C424—C425—S421	112.0 (2)
C3—N2—N1	105.3 (2)	C424—C425—H425	124.0
N2—C3—C4	111.7 (2)	S421—C425—H425	124.0
N2—C3—C31	120.5 (2)	C442—C441—C446	116.2 (3)
C4—C3—C31	127.7 (2)	C442—C441—C424	121.8 (3)
C5—C4—C3	104.5 (2)	C446—C441—C424	122.0 (3)
C5—C4—C43	127.4 (3)	C443—C442—C441	122.0 (3)
C3—C4—C43	128.1 (2)	C443—C442—H442	119.0
C4—C5—N1	108.7 (2)	C441—C442—H442	119.0
C4—C5—O51	128.6 (2)	C442—C443—C444	120.1 (3)
N1—C5—O51	122.7 (2)	C442—C443—H443	119.9
C12—C11—C16	120.1 (2)	C444—C443—H443	119.9
C12—C11—N1	121.3 (2)	C445—C444—C443	119.3 (3)
C16—C11—N1	118.6 (2)	C445—C444—Br44	120.4 (3)
C11—C12—C13	119.4 (3)	C443—C444—Br44	120.3 (3)
C11—C12—H12	120.3	C446—C445—C444	120.1 (3)
C13—C12—H12	120.3	C446—C445—H445	120.0
C14—C13—C12	120.7 (3)	C444—C445—H445	120.0
C14—C13—H13	119.7	C445—C446—C441	122.3 (3)
C12—C13—H13	119.7	C445—C446—H446	118.9
C13—C14—C15	119.5 (3)	C441—C446—H446	118.9
C13—C14—H14	120.2	C452—S451—C455	92.0 (3)
C15—C14—H14	120.2	C453—C452—C45	124.7 (4)
C14—C15—C16	120.9 (3)	C453—C452—S451	112.1 (3)
C14—C15—H15	119.6	C45—C452—S451	123.2 (3)
C16—C15—H15	119.6	C452—C453—C454	111.1 (6)
C11—C16—C15	119.4 (3)	C452—C453—H453	124.4
C11—C16—H16	120.3	C454—C453—H453	124.4
C15—C16—H16	120.3	C455—C454—C453	112.2 (5)
C3—C31—H31A	109.5	C455—C454—H454	123.9
C3—C31—H31B	109.5	C453—C454—H454	123.9
H31A—C31—H31B	109.5	C454—C455—S451	112.5 (4)
C3—C31—H31C	109.5	C454—C455—H455	123.8
H31A—C31—H31C	109.5	S451—C455—H455	123.8
H31B—C31—H31C	109.5	C554—C553—H553	124.5
C45—N41—N42	108.5 (3)	C555—C554—C553	111.9 (12)
C422—N42—N41	119.3 (3)	C555—C554—H554	124.0
C422—N42—C43	126.7 (3)	C553—C554—H554	124.0
N41—N42—C43	114.0 (2)	C554—C555—S551	112.0 (15)
N42—C43—C4	113.5 (2)	C554—C555—H555	124.0
N42—C43—C44	100.3 (2)	S551—C555—H555	124.0
C4—C43—C44	115.3 (2)	C5—O51—C51	115.95 (19)
N42—C43—H43	109.1	C56—C51—C52	119.1 (2)
C4—C43—H43	109.1	C56—C51—O51	123.4 (2)
C44—C43—H43	109.1	C52—C51—O51	117.5 (2)
C45—C44—C43	103.5 (2)	C53—C52—C51	120.9 (3)
C45—C44—H44A	111.1	C53—C52—Cl52	119.7 (2)
C43—C44—H44A	111.1	C51—C52—Cl52	119.4 (2)
C45—C44—H44B	111.1	C54—C53—C52	118.8 (3)
C43—C44—H44B	111.1	C54—C53—H53	120.6
H44A—C44—H44B	109.0	C52—C53—H53	120.6
N41—C45—C452	121.5 (3)	C55—C54—C53	120.7 (3)
N41—C45—C44	113.6 (3)	C55—C54—Cl54	119.5 (2)
C452—C45—C44	124.8 (3)	C53—C54—Cl54	119.8 (2)
C425—S421—C422	87.62 (16)	C54—C55—C56	120.3 (3)
N423—C422—N42	123.9 (3)	C54—C55—H55	119.8
N423—C422—S421	116.5 (2)	C56—C55—H55	119.8
N42—C422—S421	119.6 (3)	C51—C56—C55	120.1 (3)
C422—N423—C424	109.9 (3)	C51—C56—H56	119.9
C425—C424—N423	113.9 (3)	C55—C56—H56	119.9
			
C5—N1—N2—C3	−0.4 (3)	N42—C422—N423—C424	−178.4 (2)
C11—N1—N2—C3	−176.7 (2)	S421—C422—N423—C424	1.8 (3)
N1—N2—C3—C4	−0.1 (3)	C422—N423—C424—C425	−1.0 (3)
N1—N2—C3—C31	178.2 (2)	C422—N423—C424—C441	179.2 (2)
N2—C3—C4—C5	0.6 (3)	N423—C424—C425—S421	−0.1 (3)
C31—C3—C4—C5	−177.6 (2)	C441—C424—C425—S421	179.6 (2)
N2—C3—C4—C43	−178.6 (2)	C422—S421—C425—C424	0.9 (2)
C31—C3—C4—C43	3.2 (4)	C425—C424—C441—C442	179.8 (3)
C3—C4—C5—N1	−0.8 (3)	N423—C424—C441—C442	−0.5 (4)
C43—C4—C5—N1	178.3 (2)	C425—C424—C441—C446	0.8 (4)
C3—C4—C5—O51	177.3 (2)	N423—C424—C441—C446	−179.5 (2)
C43—C4—C5—O51	−3.5 (4)	C446—C441—C442—C443	−0.5 (4)
N2—N1—C5—C4	0.8 (3)	C424—C441—C442—C443	−179.6 (3)
C11—N1—C5—C4	176.5 (2)	C441—C442—C443—C444	0.5 (5)
N2—N1—C5—O51	−177.4 (2)	C442—C443—C444—C445	−0.1 (5)
C11—N1—C5—O51	−1.7 (4)	C442—C443—C444—Br44	178.1 (2)
C5—N1—C11—C12	22.9 (4)	C443—C444—C445—C446	−0.3 (5)
N2—N1—C11—C12	−161.7 (2)	Br44—C444—C445—C446	−178.5 (2)
C5—N1—C11—C16	−157.7 (3)	C444—C445—C446—C441	0.3 (5)
N2—N1—C11—C16	17.7 (3)	C442—C441—C446—C445	0.1 (4)
C16—C11—C12—C13	1.5 (4)	C424—C441—C446—C445	179.2 (3)
N1—C11—C12—C13	−179.1 (2)	N41—C45—C452—C453	−175.4 (5)
C11—C12—C13—C14	−1.0 (4)	C44—C45—C452—C453	3.4 (6)
C12—C13—C14—C15	0.0 (4)	N41—C45—C452—S451	5.6 (4)
C13—C14—C15—C16	0.5 (5)	C44—C45—C452—S451	−175.5 (2)
C12—C11—C16—C15	−1.0 (4)	C455—S451—C452—C453	−0.1 (4)
N1—C11—C16—C15	179.6 (2)	C455—S451—C452—C45	179.0 (3)
C14—C15—C16—C11	0.0 (4)	C45—C452—C453—C454	−179.4 (4)
C45—N41—N42—C422	179.6 (2)	S451—C452—C453—C454	−0.3 (6)
C45—N41—N42—C43	0.5 (3)	C452—C453—C454—C455	0.6 (7)
C422—N42—C43—C4	55.9 (4)	C453—C454—C455—S451	−0.6 (7)
N41—N42—C43—C4	−125.0 (3)	C452—S451—C455—C454	0.4 (5)
C422—N42—C43—C44	179.5 (2)	C553—C554—C555—S551	8 (16)
N41—N42—C43—C44	−1.4 (3)	C4—C5—O51—C51	−84.1 (3)
C5—C4—C43—N42	63.0 (4)	N1—C5—O51—C51	93.9 (3)
C3—C4—C43—N42	−118.0 (3)	C5—O51—C51—C56	−17.9 (3)
C5—C4—C43—C44	−51.9 (4)	C5—O51—C51—C52	162.1 (2)
C3—C4—C43—C44	127.1 (3)	C56—C51—C52—C53	0.5 (4)
N42—C43—C44—C45	1.7 (2)	O51—C51—C52—C53	−179.5 (2)
C4—C43—C44—C45	124.0 (2)	C56—C51—C52—Cl52	−179.80 (19)
N42—N41—C45—C452	179.8 (2)	O51—C51—C52—Cl52	0.2 (3)
N42—N41—C45—C44	0.8 (3)	C51—C52—C53—C54	0.1 (4)
C43—C44—C45—N41	−1.7 (3)	Cl52—C52—C53—C54	−179.63 (19)
C43—C44—C45—C452	179.3 (2)	C52—C53—C54—C55	−0.5 (4)
N41—N42—C422—N423	−177.2 (2)	C52—C53—C54—Cl54	179.2 (2)
C43—N42—C422—N423	1.9 (4)	C53—C54—C55—C56	0.3 (4)
N41—N42—C422—S421	2.6 (3)	Cl54—C54—C55—C56	−179.3 (2)
C43—N42—C422—S421	−178.31 (19)	C52—C51—C56—C55	−0.7 (4)
C425—S421—C422—N423	−1.6 (2)	O51—C51—C56—C55	179.4 (2)
C425—S421—C422—N42	178.6 (2)	C54—C55—C56—C51	0.3 (4)
